# Non-Alkaloid Cholinesterase Inhibitory Compounds from Natural Sources

**DOI:** 10.3390/molecules26185582

**Published:** 2021-09-14

**Authors:** Alfred Ngenge Tamfu, Selcuk Kucukaydin, Balakyz Yeskaliyeva, Mehmet Ozturk, Rodica Mihaela Dinica

**Affiliations:** 1 School of Chemical Engineering and Mineral Industries, University of Ngaoundere, 454 Ngaoundere, Cameroon; 2 Department of Chemistry, Mugla Sitki Kocman University, Mugla 48000, Turkey; balakyzyes@gmail.com (B.Y.); mehmetozturk@mu.edu.tr (M.O.); 3 Department of Medical Services and Techniques, Koycegiz Vocational School of Health Services, Mugla Sitki Kocman University, Mugla 48800, Turkey; selcukkucukaydin@gmail.com; 4 Faculty of Chemistry and Chemical Technology, Al-Farabi Kazakh National University, Almaty 050040, Kazakhstan; 5 Department of Chemistry, Physics and Environment, Faculty of Sciences and Environment, Dunarea de Jos University, 47 Domneasca Str., 800008 Galati, Romania

**Keywords:** Alzheimer’s disease, cholinesterase inhibitors, terpenoids, phenolic compounds, coumarins

## Abstract

Alzheimer’s disease (AD) is a severe neurodegenerative disorder of different brain regions accompanied by distresses and affecting more than 25 million people in the world. This progressive brain deterioration affects the central nervous system and has negative impacts on a patient’s daily activities such as memory impairment. The most important challenge concerning AD is the development of new drugs for long-term treatment or prevention, with lesser side effects and greater efficiency as cholinesterases inhibitors and the ability to remove amyloid-beta(Aβ) deposits and other related AD neuropathologies. Natural sources provide promising alternatives to synthetic cholinesterase inhibitors and many have been reported for alkaloids while neglecting other classes with potential cholinesterase inhibition. This review summarizes information about the therapeutic potential of small natural molecules from medicinal herbs, belonging to terpenoids, coumarins, and phenolic compounds, and others, which have gained special attention due to their specific modes of action and their advantages of low toxicity and high efficiency in the treatment of AD. Some show superior drug-like features in comparison to synthetic cholinesterase inhibitors. We expect that the listed phytoconstituents in this review will serve as promising tools and chemical scaffolds for the discovery of new potent therapeutic leads for the amelioration and treatment of Alzheimer’s disease.

## 1. Introduction

The research of novel drug candidates has shown that natural products such as plant extracts and plant-originated compounds have enormous potential to become drug leads with neuroprotective activity. Several non-alkaloid phytochemicals have been obtained from natural sources, including terpenoids, coumarins, flavonoids, and other phenolic compounds which have beneficial neuroprotective properties particularly in cholinesterase inhibition hence, they are potential drug candidates for the treatment of Alzheimer’s disease (AD). Alzheimer’s disease (AD), one of the leading causes of dementia, is an overwhelming neurodegenerative disease that particularly affects brain function, resulting in memory loss and impairment of language, emotional disturbance, personality changes, depression, behavioral problems, and judgment capacity [1,2]. Besides dementia, it is a major cause of death amongst old people. In the brains of Alzheimer’s disease (AD) patients, key neuropathological features of pathological protein deposits such as insoluble amyloid-β (Aβ peptides which form senile plaques) and hyperphosphorylated tau (which aggregates into NFTs) have been revealed [3]. It was reported that 35.6 million individuals suffered from AD in 2010, over 44 million people had dementia in 2013, and that the number will increase regularly to around 115 to 135 million individuals by 2050 [4,5]. The major physiological evidence of AD involves the degradation of cholinergic neurons and reduction in acetylcholine. 

Cholinergic neurotransmission is terminated by two cholinesterases acetylcholinesterase (AChE) and butyrylcholinesterase (BChE), which play an essential role in the hydrolysis of ACh [6]. According to the cholinergic hypothesis, memory impairment in Alzheimer’s disease is due to the deficit of cholinergic function in the brain, thereby, reducing hippocampal and cortical levels of the neurotransmitter acetylcholine (ACh) and associated enzyme choline transferase [7,8]. In the healthy brain, acetylcholinesterase (AChE) is the most important enzyme regulating the level of ACh, while butyrylcholinesterase (BChE) plays a minor role [1]. It is therefore expected that if the hydrolysis of ACh by AChE and BChE is inhibited in the brain of an AD patient, the amount of ACh in the synapse will be significantly increased and the neurotransmission mechanism will be more fluid [9]. For this reason, acetylcholinesterase (AChE) and butyrylcholinesterase (BuChE) inhibitors such as galantamine, donepezil, and rivastigmine are used in the management of AD, and the inhibition of the two types of cholinesterase enzymes (AChE and BuChE) as remedial for such treatment [10]. However, the high cost, non-selectivity, limited efficacy, poor bioavailability, and adverse cholinergic side effects in the periphery, such as nausea, vomiting, diarrhea, dizziness, gastro-intestinal disorders, moderate to low effectiveness, short half-life, and hepatotoxicity are the several limitations of these drugs [11]. These reasons have prompted the search for newer molecules from natural products by researchers worldwide because cholinesterase inhibitors are known to occur in plants used traditionally for failing memory and other cognitive declines associated with age [12]. For example, galantamine, physostigmine, and huperzine A have been isolated from *Galanthus nivalis*, Physostigma venenosum, and *Huperzia serrata*, respectively, and clinically used for AD symptomatic management [13].

Alkaloids include a high number of compounds with anticholinesterases, though some terpenes, coumarins, and lignans have been shown to have this activity. Recently, research has targeted alkaloid compounds as potent anticholinesterase compounds and little attention has been given to other classes. In this review, we report a representative update of terpenoids, phenolic, and coumarin compounds with their AChE and BChE inhibitory potentials according to reports from 2009 to 2021.

## 2. Natural Non-Alkaloid Cholinesterase Inhibitors

Alzheimer’s disease (AD) is the most common form of dementia mostly in old people, characterized by low acetylcholine levels and oxidative stress, involving progressive neurodegeneration with the formation of amyloid-β deposits in the brain. The number of individuals suffering from this disease and its related neuropathologies has been increasing over the years and a majority of the patients are old people. A proper strategy to overcome AD is by the inhibition of cholinesterase enzymes which helps to increase acetylcholine levels in the brain which is necessary for neurotransmission, memory, reasoning, and other cognitive activities. Though synthetic cholinesterase inhibitors, including rivastigmine, donepezil, and galantamine are usually employed as a remedy to AD, there is a growing interest in the search for new cholinesterase inhibitors from natural sources due to the drawbacks of synthetic ones, and most non-alkaloid natural anticholinesterase compounds are terpenoids, phenolic compounds, and coumarins, amongst others.

**Figure 1:**
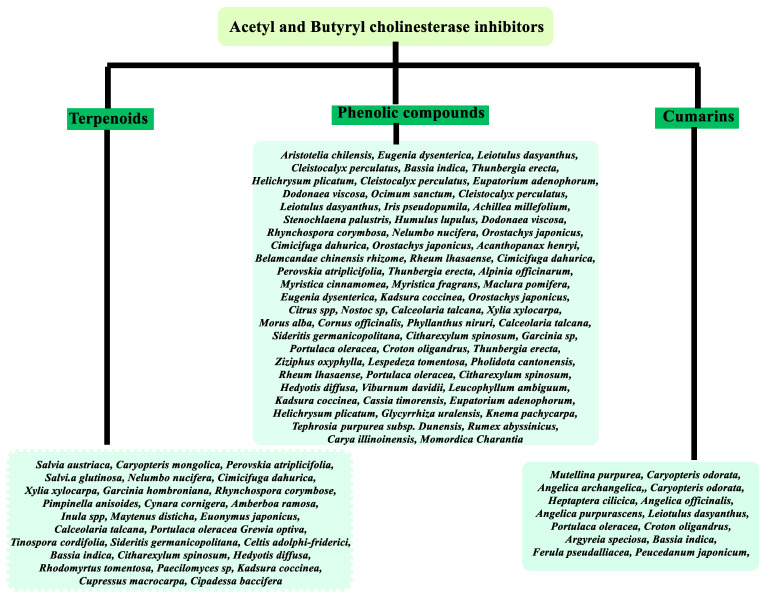
Non-alkaloid cholinesterases inhibitors and their natural sources.

The Supplementary Material (Tables S1–S4) gives the representative non-alkaloid naturally occurring compounds with useful anticholinesterase properties obtained mainly from plants as summarized below (Figure 1). Figure 1 gives a vivid indication of sources of starting material for cholinesterase inhibitory compounds and drugs. The compounds obtained from these plants have been explored to target pathological features in neurodegenerative diseases such as AD and can be also used as a starting point to design a new library of potent derivatives.

## 3. Discussion

### 3.1. Terpenoids

Several terpenoids from natural sources have been reported as cholinesterase inhibi-tors [14,15,16,17,18,19,20,21,22,23,24,25,26,27,28,29,30,31,32,33,34,35,36,37,38,39,40,41,42]. The compounds **1**–**11**, as shown in Supplementary Material (Table S1), are of the Abietane-type diterpene skeletons isolated from *Salvia austriaca*, *Salvia glutinosa*, *Caryopteris mongolica,* and *Perovskia atriplicifolia* [14,15,16]. Between compounds **2**–**7**, there is an -OH group on the side chain, except for compound **5** which has a methoxy (CH_3_O-) group on the side chain and has the highest AChE inhibition activity. The high activity could be due to the presence of this methoxy group. In the same way, compound **2** has good activity and possesses a methoxy group on ring C and has an IC_50_ of 27.9 ± 5.2 µM compared to compound **5** with an IC_50_ of 20.8 ± 7.1 µM. Compounds **8** to **11** are miltirone derivatives though with little structural differences, there is no significant difference in their cholinesterase inhibition activity. Compounds **12**–**19** are tanshinone derivatives [16]. Compounds **16** and **17** are the most active with percentage inhibitions of 6.19 ± 3.91% and 5.55 ± 3.03%, respectively at 10 µg/mL. This could be attributed to the conjugated double bond system in ring A which is particular to these two compounds. Compound **20**, a monoterpene glycoside nuciferoside, shows very high activity with an IC_50_ value of 3.20 ± 0.22 µM [17]. Compounds **21**–**36** are cycloartanes triterpenoids isolated from *Cimicifuga dahurica* and *Nelumbo nucifera* [17,18]. Amongst them, compound **25** is the most active with a percentage inhibition of 15.8 ± 4.3% and 14.0 ± 2.6% on AChE and BChE, respectively, at 100 µM. This could possibly be attributed to the absence of the double bond in ring B of this compound. Compounds **37**–**42** are lupane type triterpenoids isolated from *Garcinia hombroniana* and *Xylia xylocarpa* and they show relatively low activities [19,20]. The oleanane triterpenoids 43, 44, and 45 isolated from *Xylia xylocarpa* and *Rhynchospora corymbose* show low activities [19,21]. The sterols 46 and 47 from *Rhynchospora corymbose* show low activities as well as the monoterpenes 48, 49, and 50 from *Pimpinella anisoides* [21,22]. Sesquiterpene lactones from 51–64 isolated from *Inula* spp., *Cynara cornigera*, and *Amberboa ramosa* show good anticholinesterase activities [23,24,25]. Compounds **58**–**61** are amberbin C, amberin, amberbin A, and amberbin B, and have high anticholinesterase activity [24]. Amongst them, those possessing sugar moieties, amberin (IC_50_ 17.5 ± 0.01 µM and 2.7 ± 0.02 µM for AChE and BChE, respectively) and amberin B (IC_50_ 0.91 ± 0.015 µM and 2.5 ± 0.15 µM for AChE and BChE, respectively) are the most active and the structural difference between them is the interchange of the positions of an acetyl group and sugar moiety. The agarofuran derivatives 62–73 isolated from *Euonymus japonicus* and *Maytenus disticha* have low activities [25,26]. The taraxaranes 74, 75, and 76, oleananes 77, 78, and 79, as well as the ursane tritepenoids 80 and 81, have relatively low activities except for compound **74** with IC_50_ values of 13.5 ± 0.95 µM and 10.6 ± 0.54 µM on AChE and BChE inhibitions [20,27,28]. Its relatively high activity could be attributed to the presence of the caffeoyl group at position 3. It can be concluded that amongst the terpenoids, sesquiterpenes are the most active compounds, especially sesquiterpene lactones.

### 3.2. Phenolic Compounds

Phenolic compounds from natural sources have shown anticholinesterase activity in several studies [34,43,44,45,46,47,48,49,50,51,52,53,54,55,56,57,58,59,60,61,62,63,64,65,66,67,68,69,70,71,72,73,74,75,76,77,78,79,80,81,82,83,84]. Phenolic compounds whose anticholinesterase activities have been reported are given in the Supplementary Material (Table S2). Compounds **1**–**19** are flavone derivatives with a double bond in ring C and a carbonyl at position 4 [34,43,44,45,46,47,48,49,50,51,52,53]. There is no observable regular pattern of variation in activity. However, compounds with no hydroxyl group on position 3 show seemingly high cholinesterase inhibition activity, for example, compounds **4** and **5**. However, compounds **16**–**19** do not have a hydroxyl group at position 3 but their activities are low and could be accounted for by the occurrence of methoxy groups on the other rings. There is an observable decrease in cholinesterase inhibition in flavones with methoxy substituents, for example, compounds **13** and **14**, and compounds **7** and **8**. This observation is not true for compounds **14** and **15** as **14** has a methoxy group on ring B but is more active than **15** without a methoxy group. This could be due to the absence of a substituent on ring B of compound **15**. Between cirsilineol (**18**) and isothymusin (**19**), an additional hydroxy group on ring A causes a decrease in cholinesterase inhibition. For the flavonoid glycosides, compounds **20**–**33** [43,45,46,49,50,54,55], those with a sugar moiety at position 7, have higher activities than the others, for example, **27**, **28**, and **29** isolated from *Achillea millefolium*. If the sugar has substituents, as is the case of **32** and **33**, the activity is further reduced. Compounds **34** to **38**, isolated from *Dodonaea viscosa*, have isoprenyl substituents but, however, show no significant difference in their activities [51]. Rather, their activities are lower than their corresponding compounds without isoprenyl substituents. Compounds **39**–**41** have acetyl groups and their BChE inhibitory activity decreases with an increase in the number of acetyl groups [21]. The phenolic acid compounds **42**–**45**, and compound **44** ferulic acid methyl ester have a good percentage of cholinesterase inhibition [17,18,57]. The presence of sugar substituents causes a decrease in cholinesterase inhibition as seen in compounds **46**–**48** [17,58], while an additional phenolic group causes an increase in cholinesterase inhibition as seen in compounds **49**–**64** [16,18,47,52,59,60,61,62]. Amongst the biphenyl compounds, **58**, **59**, **60**, and **61** isolated from *Myristica cinnamomea* have high activity, and in these compounds, the carbonyl function is adjacent to one of the phenyl groups (phenyl carbonyl). Isoflavones compounds **65**–**79** isolated from *Iris pseudopumila*, *Maclura pomifera*, and *Belamcandae chinensis rhizoma* have low activities [52,54,59,63]. Amongst them, methoxy substituents cause no significant change in cholinesterase inhibition while the presence of sugar molecules causes a decrease in this activity. For those with prenyl groups (**75**–**79**) isolated from *Maclura pomifera*, there was no observable effect due to the presence of the prenyls, but an -OH group on ring B caused an increase in activity between compounds **75** and **76**. Catechin and its derivatives **79** to **84** isolated from *Eugenia dysenterica* and *Orostachys japonicus* had no good activity and no significant difference despite structural differences except between compound **82** and **83** where the additional benzoic acid substituent increased AChE and BChE inhibition activities [40,44,57]. This observation was similar for the flavanones **85** to **89**, though the addition of sugar molecules caused an increase in AChE and BChE inhibitions in compound **90** compared to compound 85 [50,51,56,64]. The xanthones compound **95**–**101** isolated from *Garcinia mangostana* and *Belamcandae chinensis rhizoma* showed moderate to good AChE and BChE inhibition activities [19,66,67]. Evidently, an increase in the hydroxyl groups causes an increase in the cholinesterase inhibitory activity of these xanthones, while no significant difference in cholinesterase inhibition is observed for the prenyl groups. For the chalcones **102** to **105** isolated from *Humulus lupulus*, the activity decreases from compound **102** to **105** with a decrease in the number of hydroxyl (-OH) substituents [56]. Aurones **106**–**109** isolated from *Morus alba* have low activities though 109 had BChE inhibition with an IC_50_ of 7.22 ± 0.22 µM [68]. Amongst the tannin compounds **110** to **115**, isolated from *Cornus officinalis*, *Phyllanthus niruri*, and *Calceolaria talcana*, compound **114** (Isocorilagin) is the most active with an IC_50_ of 0.49 µM and 4.20 µM on AChE and BChE inhibition, respectively [28,32,36,69,70]. This could be because it is less bulky, having only three benzoyl groups as compared to compounds **112** and **113** with five benzoyl groups and **110** and **111** with four benzoyl groups. The triflavanone Garcineflavanone A and biflavonol Garcineflavonol A isolated from *Garcinia atroviridis* both showed good percentage inhibition of cholinesterase. *M. charantia* extract showed many inhibitory activities, however, ligballinol a lignan found in extract showed relatively high activity. According to previous studies, not many lignans have been reported to exhibit cholinesterase inhibitory activity [85].

### 3.3. Coumarins

Coumarins constitute another important class of cholinesterase inhibitors as seen in some scientific reports [86,87,88,89,90,91,92,93,94,95]. Compounds **1**–**6** (Table S3) isolated from *Angelica archangelica*, *Caryopteris odorata*, and *Mutellina purpurea* showed low activities, and although with slight structural differences, there is no significant difference in their activities [86,87,88,89]. Between compounds **6** and **7**, the addition of a prenyl group decreases the percentage inhibition. Compound **9**, Umbelliprenin, isolated from *Heptaptera cilicica*, shows good activity with IC_50_ values of 5.86 ± 0.030 µM and 1.10 ± 0.190 µM on AChE and BChE inhibition [90,91]. By adding hydroxyl (-OH), carboxyl (-COOH), or acetyl substituent to compounds **10**, **11**, and **12**, respectively, the percentage inhibitions increase as compared to compound **7** [90]. Subsequent addition of isoprenyl groups, as seen in compounds **13** and **14**, decreases the percentage cholinesterase inhibition [90]. This effect is illustrated with compounds **15** and **16** in which addition of one isoprenyl group to compound **15** to obtain **16** decreases AChE percentage inhibition from 11.47 ± 1.73% to 7.03 ± 2.08% and also between compound **17** and **19** where the addition of one isoprenyl decrease BChE inhibition from 51.04 ± 1.88% to 23.82 ± 2.41% [90]. For di-o prenylated coumarins, **22** with two *O*-geranyl groups and **23** with two *O*-farnesyl groups, **23** shows a higher percentage inhibition than **22**, and this could be attributed to the additional isoprene unit in **23** [90]. For the coumarins **24** to **32**, the only structural difference is on the side chain and this causes a significant difference in the cholinesterase inhibition activity of the corresponding compounds [90]. For these compounds, unsaturation in the side chain caused no significant change in the cholinesterase inhibition activity. However, compound **26** having a styryloxy group has the highest AChE inhibition percentage while compound **30** with the isobutyloxy group has the highest BChE inhibition activity. Compounds **33** to **41**, isolated from *Angelica officinalis*, *Leiotulus dasyanthus*, and *Angelica archangelica*, did not show significant activity [45,88,89,92]. The umbelliferone and its derivatives, compounds **42** to **46** isolated from *Angelica archangelica*, *Leiotulus dasyanthus*, and *Heptaptera cilicica*, showed good activity [45,88,91]. The most active umbelliferone derivatives were conferone (IC_50_ 3.31 ± 0.014 µM and 9.31 ± 0.280 µM on AChE and BChE respectively), mogoltacin (IC_50_ 1.95 ± 0.050 µM and 9.74 ± 0.003 µM on AChE and BChE respectively), and feselol (IC_50_ 1.26 ± 0.010 µM and 9.98 ± 0.240 µM on AChE and BChE respectively) and were all isolated from *Heptaptera cilicica*. It can be concluded that, in the class of coumarins, umbelliferone derivatives are the most potent cholinesterase inhibitory compounds.

Other miscellenous compounds (Table S4) have equally shown interesting acetylcho-linesterase and butyrylcholinesterase inhibitory activities [96,97,98,99,100,101,102,103,104,105,106,107,108,109,110,111,112,113,114,115,116].

### 3.4. Some Considerations on Terpenoids, Phenolic Compounds, and Coumarins as Cholinesterase Inhibitors

It is important to search for new therapies which are more effective than those currently existing, and which can both prevent neurodegenerative diseases such as AD and block the progression of these pathologies at their early stages, thereby reducing the socioeconomic costs involved in the management of AD and the patients [117]. Acetylcholine is a key neurotransmitter involved in cognitive activities, but its activity can be reduced by AChE and BChE which hydrolyze acetylcholine into choline and acetic acid causing the cholinergic neurotransmission to decrease. The development of many therapies for AD is based mainly on this cholinergic hypothesis, and the remediation of acetylcholine levels and cholinergic function in the central nervous system through the inhibition of cholinesterase enzymes (AChE and BChE) can eliminate the pathologies of AD. The classes of cholinesterase inhibitors discussed here are mainly terpenoids, phenolic compounds, and coumarins, and some of these compounds have shown high potency. In order to consider which classes are most suitable, based on the benefits and drawbacks, certain structural features of each class will be of great importance. Using chalcones as an example, it is believed that besides economical and cost-effective production, small molecular size and flexibility for modifications to improve lipophilicity necessary for blood-brain barrier permeability are important to consider for a preferred potential therapeutic candidate for AD [118]. Terpenoids are able to inhibit cholinesterases in different ways. It has been shown that 1,8-cineole, α-pinene, and camphor could inhibit AChE reversibly [119]. Certain tanshinone derivatives could be noncompetitive inhibitors of AChE and BChE in humans and are able to bind to the allosteric site of cholinesterases principally through hydrophobic interactions and also through hydrogen bonds with Tyr337 and Gly120 of AChE [120]. Carbonyl function in terpenes can bind by covalent arrangement to the free amino or sulfhydryl groups of the enzyme while phenolic hydroxyl groups bind proteins, leading to the conformational change of the enzyme [121]. Terpene alcohols and terpene hydrocarbon compounds possess identical cholinesterase inhibition while terpenoids with a ketone group exhibit stronger cholinesterase inhibition and an allylic group increases activity [121,122]. Amongst the terpenoids, monoterpenoids are the most promising because the inhibition of AChE has been shown to remedy AD by inhibiting amyloid-beta-induced neurotoxicity and also clearing it, tau-protein phosphorylation, and oxidative stress by boosting antioxidant defenses, neuroinflammation, restoration of mitochondrial function, initiation of processes with simultaneous inhibition of pro-apoptotic genes and proteins [123]. In the phenolic compounds, inhibitory activity is influenced by the position and number of hydroxyl and methoxyl groups bonded to the phenol ring, and the methoxy substitution on the phenol ring improves cholinesterase inhibitory activity and phenolic acids are capable of inhibiting the formation of amyloid β-peptide (Aβ) fibrils [124]. Phenolic compounds exert neuroprotective effects, though it is assumed that the transfer of polyphenols through the blood–brain barrier is limited, likewise, a considerable number of reports discuss the absorption and presence of phenolic acids in the brain [125]. Phenolic compounds are able to bind to the active sites of AChE or BChE resulting in the inhibition of these enzymes [126]. Aromatic ring moieties are suggested to be involved in the selection and stabilization of the positive charge of the quaternary group in the acetylcholine, and some of the flavonoids can induce modifications in the structure of cholinesterase enzymes blocking entrance into the active site and those with free OH- groups are also more potent than glycosylated ones. Phenolic compounds structurally similar to caffeic acid are capable of fitting into the gorge of the active site of AChE and are more potent [127]. In coumarins, it has been shown that those with larger substituents at position 7 have a higher inhibitory effect than those with small substituent groups at the same position, and equally, compounds that contain a coumarin nucleus and a long-chain substituent with some phenyl and aryl/benzyl-piperazines groups can be more potent inhibitors of cholinesterase [128,129]. The anticholinesterase activity of coumarins is mostly dependent on their binding ability on the enzyme, and this activity is greatly improved in the scaffolds with some cholinesterase inhibitory drugs [129]. Moreover, the structure of coumarins is highly modifiable through chemical means, thereby presenting them as suitable starting materials for the synthesis of drugs. Many cholinesterase drugs with a coumarin nucleus have been reported, making coumarin a priority pharmacophore for cholinesterase inhibitors. In a study in which 36 isolated compounds were classified and discussed according to their anti-AChE pharmacological potency, phenolic compounds and flavonoids were mostly found in the low activity zone of natural acetylcholinesterase inhibitors according to their ability to bind to the active site of acetylcholinesterase [130]. Coumarins and terpenoids occupied zones indicated as moderate to high activity and capacity of binding to the active site of acetylcholinesterase [130]. 

However, preclinical, clinical safety, selectivity, and toxicity of these compounds are not established, and classification of these compounds based on their benefits and drawbacks will be controversial and non-conclusive. However, a common point seems to be the overall size of the compound which should generally be small so as to be able to cross the blood–brain barrier and exerts its function.

## 4. Conclusions

Neurodegenerative disease is a generic term applied to a variety of conditions arising from a chronic breakdown and deterioration of the central nervous system (CNS) neurons. Many of these diseases exist, but Alzheimer’s disease (AD) is the most prevalent. Alzheimer’s disease (AD) patients present a progressive loss of cholinergic synapses in the brain regions associated with a decrease in the acetylcholine (ACh), a neurotransmitter, which appears to be a critical element in the development of dementia. Hence, AD and other forms of dementia could be treated by the use of agents that restore the level of acetylcholine through the inhibition of both major forms of cholinesterase: acetylcholinesterase (AChE) and butyrylcholinesterase (BChE). Loizzo and co-workers postulated that AD causes and progression involves four relevant pathogenic events: primary events (genetic alterations, neuronal apoptosis-like processes leading to premature neuronal death and brain dysfunction), secondary events (β-amyloid deposition in senile plaques and brain vessels, neurofibrillary tangles due to the hyperphosphorylation of tau proteins, synaptic loss), tertiary events (neurotransmitter deficits, neurotrophic alterations, neuroimmune dysfunction, neuroinflammatory processes), and quaternary events (accelerated neuronal death due to excitotoxic reactions, alterations in calcium homeostasis, free radical formation, cerebrovascular dysfunction) [117]. The potential use of natural products in the treatment of neurodegenerative disorders has also been successfully demonstrated in the field of AD, and also to treat other forms of dementia including vascular dementia, Parkinson’s dementia, dementia/Lewy body, and cognitive symptoms associated with multiple sclerosis and Down syndrome. However, much attention is focused on alkaloids while little is given to phenolics, terpenoids, and coumarins, and this review gives an update of representative non-alkaloid compounds with anticholinesterase activity. 

## Supplementary Materials

The following are available as supplementary material online; Table S1: Terpenoids as Acetyl and Butyryl cholinesterase inhibitors, Table S2: Phenolic compounds as Acetyl and Butyryl cho-linesterase inhibitors, Table S3: Coumarins as Acetyl and Butyryl cholinesterase inhibitors and Table S4: Other Acetyl and Butyryl cholinesterase inhibitors.
