# Supplementary material for: Non-Alkaloid Cholinesterase Inhibitory Compounds from Natural Sources
Source: Molecules. 2021 Sep 14;26(18):5582. doi: 10.3390/molecules26185582 (PMC8472022; doi:10.3390/molecules26185582)
Supplement: Supplementary file 1 [file molecules-26-05582-s001.zip › molecules-1372198-supplementary.pdf]

molecules

MDPI

Review

Non-Alkaloid Cholinesterase Inhibitory Compounds from Natural Sources

Alfred Ngenge Tamfu [1,4*], Selcuk Kucukaydin [2], Balakyz Yeskaliyeva [3,4], Mehmet Ozturk [4] and Rodica Mihaela Dinica [5*]

[1] School of Chemical Engineering and Mineral Industries, University of Ngaoundere, 454 Ngaoundere, Cameroon; macntamfu@yahoo.co.uk
[2] Department of Medical Services and Techniques, Koycegiz Vocational School of Health Services, Mugla Sitki Kocman University, 48800 Mugla, Turkey; selcukkucukaydin@gmail.com
[3] Faculty of Chemistry and Chemical Technology, Al-Farabi Kazakh National University, Almaty 050040, Kazakhstan; balakyzyes@gmail.com
[4] Department of Chemistry, Mugla Sitki Kocman University, 48000 Mugla, Turkey; mehmetozturk@mu.edu.tr
[5] Dunarea de Jos University, Faculty of Sciences and Environment, Department of Chemistry, Physics and Environment, 47 Domneasca Str., 800008, Galati, Romania; rodica.dinica@ugal.ro

* Correspondence: macntamfu@yahoo.co.uk (A.N.T.); rodica.dinica@ugal.ro (R.M.D.); Tel.: +237-675590353 (A.N.T.); +33-6130-251 (R.M.D.)

Supplementary Material

Table S1: Terpenoids as Acetyl and Butyryl cholinesterase inhibitors

No	Name	Structure	Plant source	IC_{50} or %inh (conc.)		Reference
				AChE	BuChE	
1.	Taxodione	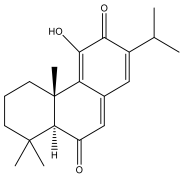	*Salvia austriaca*	54.84 µg/mL (IC_{50})	195.9 µg/mL (IC_{50})	[14]
2.	(5S,10S,16R)-11,16-dihydroxy-12-methoxy-17(15→16)-abeoabieta-8,11,13-trien -7-one	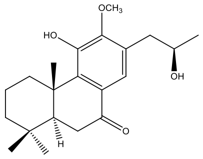	*Caryopteris mongolica*	27.9 ± 5.2 µM (IC_{50})	73.8 ± 1.7 µM (IC_{50})	[15]
3.	(5S,10S,16R)-11,12,16-trihydroxy-17(15 →16)-abeo-abieta-8,11,13-trien-7-one	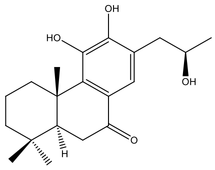	*Caryopteris mongolica*	64.6 ± 5.9 µM (IC_{50})	>100 µM (IC_{50})	[15]

4.	(5S,10S,16R)-11,12,16-trihydroxy-17(15 →16),18(4 → 3)-di-abeo-abieta -3,8,11,13-tetraen-7-one	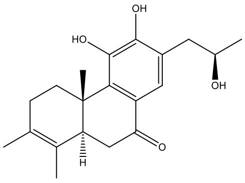	*Caryopteris mongolica*	38.4 ± 10.8 μM (IC_{50})	98.4 ± 1.1 μM (IC_{50})	[15]
5.	(5S,10S,16R)-11,12-dihydroxy-16-methoxy-17(15→16),18(4→3)-diabeo-abieta -3,8,11,13 -tetraen-7-one	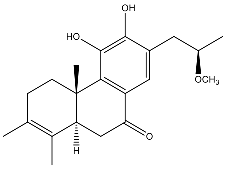	*Caryopteris mongolica*	20.8 ± 7.1 μM (IC_{50})	>100 μM (IC_{50})	[15]
6.	(15R)-cyrtophyllone B	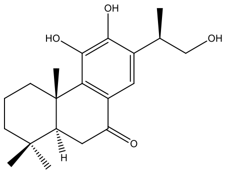	*Caryopteris mongolica*	55.7 ± 7.4 μM (IC_{50})	93.2 ± 6.2 μM (IC_{50})	[15]
7.	Incanone	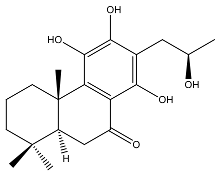	*Caryopteris mongolica*	87.5 ± 12.3 μM (IC_{50})	>100 μM (IC_{50})	[15]
8.	Arucadiol	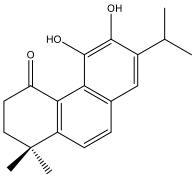	*Perovskia atriplicifolia*	-	91.97 ± 0.08% (10 μg/mL)	[16]
9.	Miltirone	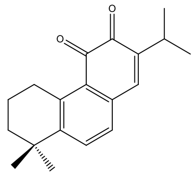	*Perovskia atriplicifolia*	-	98.36 ± 0.89% (10 μg/mL)	[16]
10.	1-Oxomiltirone	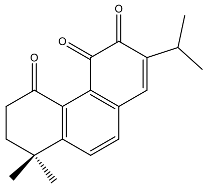	*Perovskia atriplicifolia*	11.08 ± 0.38% (10 μg/mL)	77.45 ± 1.97% (10 μg/mL)	[16]
11.	1,2-Didehydromiltirone	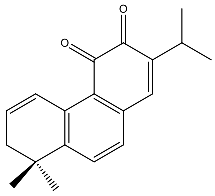	*Perovskia atriplicifolia*	10.08 ± 2.22% (10 μg/mL)	97.36 ± 2.78 % (10 μg/mL)	[16]

12.	Tanshinone IIa	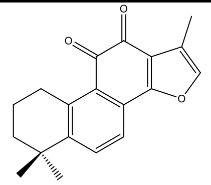	*Salvia glutinosa*	45.16 ± 5.49 % (10 μg/mL)	97.41 ± 0.23 % (10 μg/mL)	[16]
13.	Cryptotanshinone	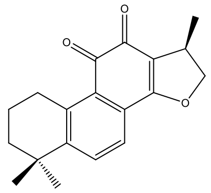	*Perovskia atriplicifolia*	28.95 ± 1.74 % (10 μg/mL)	74.37 ± 3.57% (10 μg/mL)	[16]
14.	1,2-Didehydro-tanshinone IIa	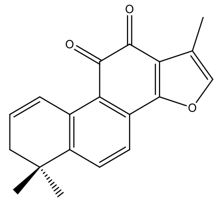	*Perovskia atriplicifolia*	41.88 ± 2.05 % (10 μg/mL)	67.65 ± 1.88 % (10 μg/mL)	[16]
15.	15,16-Dihydro-tanshinone	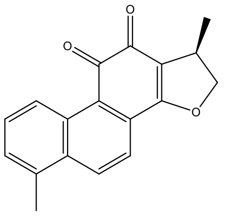	*Salvia glutinosa*	65.17 ± 1.39 % (10 μg/mL)	94.88 ± 1.88 % (10 μg/mL)	[16]
16.	Tanshinone I	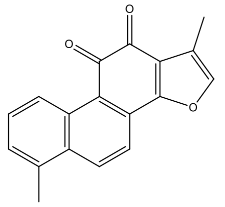	*Salvia glutinosa*	6.19 ± 3.91 % (10 μg/mL)	85.84 ± 4.15 % (10 μg/mL)	[16]
17.	Isotanshinone II	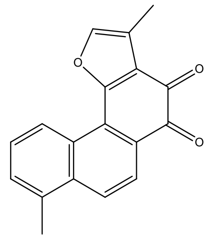	*Salvia glutinosa*	5.55 ± 3.03 % (10 μg/mL)	77.81 ± 1.45 % (10 μg/mL)	[16]
18.	1(S)-OH-Tanshinone IIa	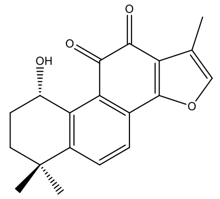	*Salvia glutinosa*	36.32 ± 1.85 % (10 μg/mL)	64.50 ± 1.50 % (10 μg/mL)	[16]
19.	1β-OH-Crypto-tanshinone	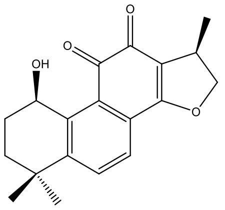	*Perovskia atriplicifolia*	17.70 ± 3.86 % (10 μg/mL)	93.15 ± 2.31 % (10 μg/mL)	[16]

20.	Nuciferoside	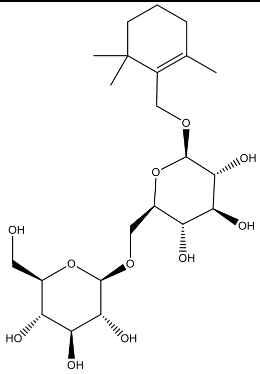	*Nelumbo nucifera*	3.20±0.22 μM (IC_{50})	83.06±0.80 μM (IC_{50})	[17]
21.	cycloartenol	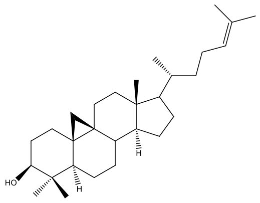	*Nelumbo nucifera*	11.89±1.91 μM (IC_{50})	13.93±1.65 μM (IC_{50})	[17]
22.	7,8-didehydrocimigenol	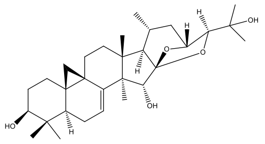	*Cimicifuga dahurica*	43.1 ± 5.6 % (100 μM)	53.6 ± 0.9 % (100 μM)	[18]
23.	24-epi-24-O-acetyl-7,8-didehydroshengmanol	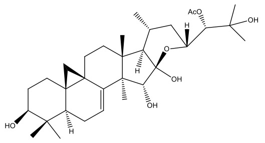	*Cimicifuga dahurica*	53.7 ± 1.6 % (100 μM)	57.3 ± 0.3 % (100 μM)	[18]
24.	25-triepoxy-12β-acetoxy-3β,26-dihydroxy-9,19-cyclolanost-7-ene	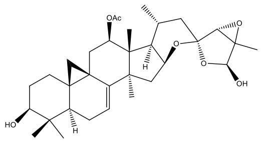	*Cimicifuga dahurica*	52.0 ± 0.1 % (100 μM)	41.0 ± 0.1 % (100 μM)	[18]
25.	25-O-acetyl-7,8-didehydrocimigenol	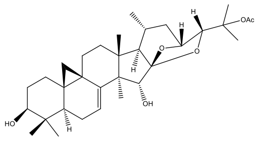	*Cimicifuga dahurica*	55.4 ± 2.6 % (100 μM)	18.0 ± 0.4 % (100 μM)	[18]
26.	25-anhydro-7,8-didehydrocimigenol	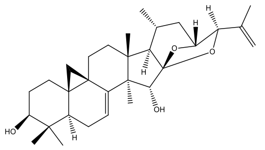	*Cimicifuga dahurica*	48.6 ± 0.6 % (100 μM)	51.0 ± 1.9 % (100 μM)	[18]
27.	24-epi-7,8-didehydrocimigenol	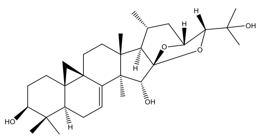	*Cimicifuga dahurica*	31.8 ± 0.5 % (100 μM)	41.1 ± 2.2 % (100 μM)	[18]
28.	25-O-acetylcimigenol	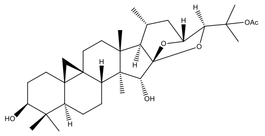	*Cimicifuga dahurica*	15.8 ± 4.3 % (100 μM)	14.0 ± 2.6 % (100 μM)	[18]

29.	24-epi-24-O-acetyl-7,8-didehydroshengmanol	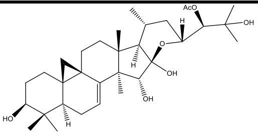	*Cimicifuga dahurica*	28.0 ± 4.0 % (100 µM)	18.6 ± 0.3 % (100 µM)	[18]
30.	25-anhydrocimigenol	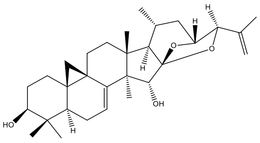	*Cimicifuga dahurica*	42.8 ± 0.8 % (100 µM)	44.9 ± 1.1 % (100 µM)	[18]
31.	25-O-acetyl-7,8-didehydrocimigenol 3-O-β-D-xylopyranoside	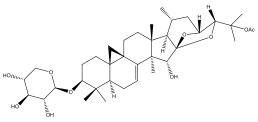	*Cimicifuga dahurica*	24.9 ± 6.2 % (100 µM)	55.0 ± 0.7 % (100 µM)	[18]
32.	25-anhydrocimigenol- 3-O-β-D-xylopyranoside	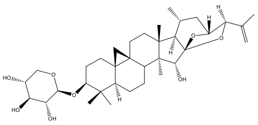	*Cimicifuga dahurica*	37.1 ± 0.5 % (100 µM)	60.7 ± 0.6 % (100 µM)	[18]
33.	24-epi-7,8-didehydrocimigenol-3-O-β-D-xylopyranoside	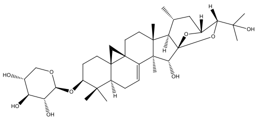	*Cimicifuga dahurica*	19.0 ± 2.5 % (100 µM)	62.0 ± 0.8 % (100 µM)	[18]
34.	3-O-β-D-xylopyranosyl-24S,25-dihydroxy-15-oxo-acta-(16R,23R)-16,23-monoxoside	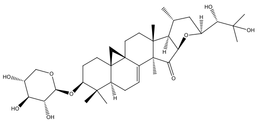	*Cimicifuga dahurica*	47.1 ± 1.6 % (100 µM)	55.4 ± 0.8 % (100 µM)	[18]
35.	Cimiricaside A	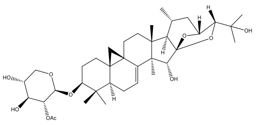	*Cimicifuga dahurica*	23.1 ± 6.8 % (100 µM)	54.4 ± 1.3 % (100 µM)	[18]
36.	7,8-didehydro-25-anhydrocimigenol-3-O-β-D-xylopyranoside	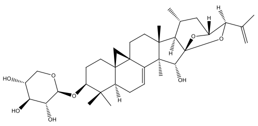	*Cimicifuga dahurica*	21.5 ± 1.7 % (100 µM)	38.5 ± 0.1 % (100 µM)	[18]
37.	(3β)-hopan-3-ol-28,22-olide	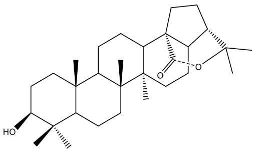	*Xylia xylocarpa*	79.5 ± 1.1 µM (IC_{50})	>100 µM (IC_{50})	[19]
38.	lupeol	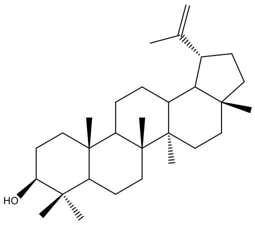	*Xylia xylocarpa*	75.7 ± 3.1 µM (IC_{50})	>100 µM (IC_{50})	[19]

39.	betulin	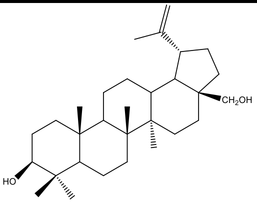	*Xylia xylocarpa*	93.4 ± 2.2 μM (IC_{50})	–	[19]
			Garcinia hombroniana	28.5 ± 0.78 (IC_{50})	-	[20]
40.	28-norlup-20(29)-ene-3β-hydroxy-17β-hydroperoxide	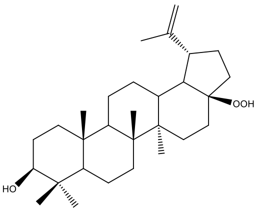	*Xylia xylocarpa*	83.9 ± 0.6 μM (IC_{50})	>100 μM (IC_{50})	[19]
41.	Betulinic acid	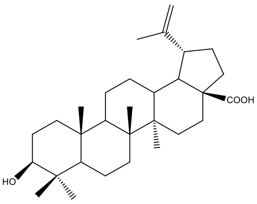	*Xylia xylocarpa*	62.0 ± 2.2 μM (IC_{50})	–	[19]
			Garcinia hombroniana	24.2 ± 0.99 μM (IC_{50})	19.1 ± 1.33 μM (IC_{50})	[20]
42.	betulonic acid	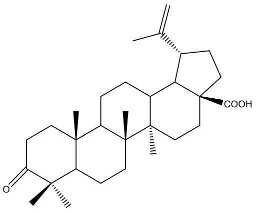	*Xylia xylocarpa*	94.6 ± 1.5 μM (IC_{50})	>100 μM (IC_{50})	[19]
43.	oleanolic acid	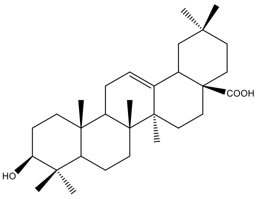	*Xylia xylocarpa*	84.9 ± 1.2 μM (IC_{50})	>100 μM (IC_{50})	[19]
44.	3β-formyloxy-l8α-oleanano-28,19β-lactone	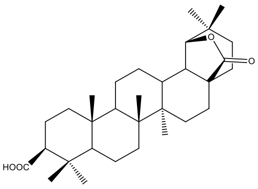	*Xylia xylocarpa*	86.5 ± 0.6 μM (IC_{50})	>100 μM (IC_{50})	[19]
45.	oleanane 3-(3′R-hydroxy)-hexadecanoate	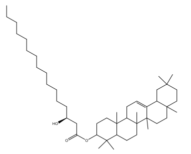	*Rhynchospora corymbosa*	-	66.49 ± 0.12 μM (IC_{50})	[21]
46.	dendrotriol	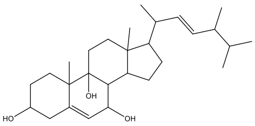	*Rhynchospora corymbosa*	-	43.43 ± 0.47 μM (IC_{50})	[21]
47.	(24R)-24-ethyl-5α-cholestane-3β,5,6β-triol	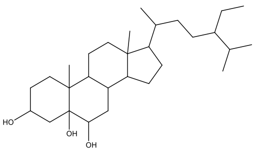	*Rhynchospora corymbosa*	-	79.44 ± 0.16 μM (IC_{50})	[21]

48.	(+)-Limonene	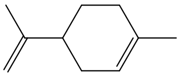	*Pimpinella anisoides*	225.9 ± 1.3 μg/mL (IC_{50})	456.2 ± 5.6 μg/mL (IC_{50})	[22]
49.	trans-Anethole	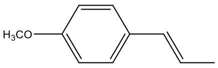	*Pimpinella anisoides*	134.7 ± 2.1 μg/mL (IC_{50})	209.6 ± 2.4 μg/mL (IC_{50})	[22]
50.	(+)-Sabinene	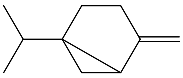	*Pimpinella anisoides*	176.5 ± 2.8 μg/mL (IC_{50})	218.6 ± 3.5 μg/mL (IC_{50})	[22]
51.	Cornigeraline	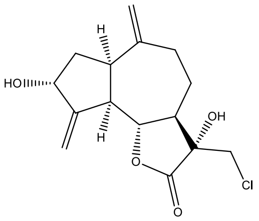	*Cynara cornigera*	20.5 μM (IC_{50})	-	[23]
52.	Solstitalin	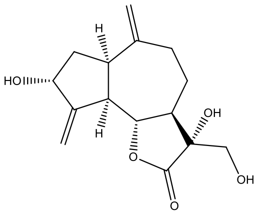	*Cynara cornigera*	35.8 μM (IC_{50})	-	[23]
53.	3-hydroxy-grosheimin	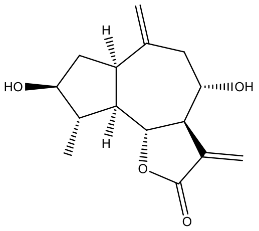	*Cynara cornigera*	30.5 μM (IC_{50})	-	[23]
54.	grosheimin	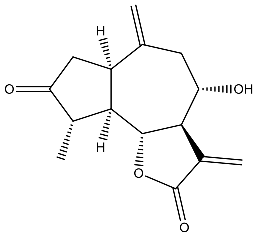	*Cynara cornigera*	61.8 μM (IC_{50})	-	[23]
55.	solstitalin A	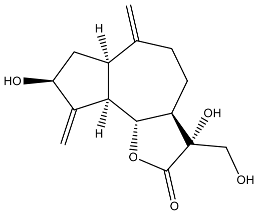	*Cynara cornigera*	25.7 μM (IC_{50})	-	[23]

56.	13-chlorosolstitialine	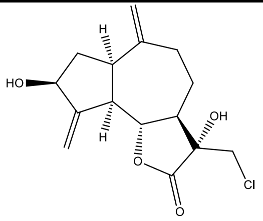	*Cynara cornigera*	62.1 μM (IC_{50})	-	[23]
57.	cyanaropicrin	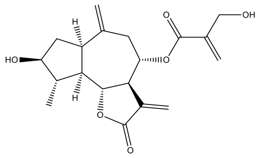	*Cynara cornigera*	31.3 μM (IC_{50})	-	[23]
58.	Amberbin C	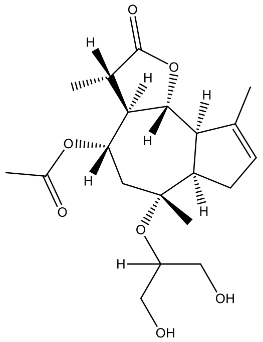	*Amberboa ramosa*	1.1 ± 0.08 μM (IC_{50})	17.9 ± 0.05 μM (IC_{50})	[24]
59.	Amberin	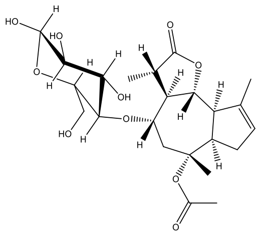	*Amberboa ramosa*	17.5 ± 0.01 μM (IC_{50})	2.7 ± 0.02 μM (IC_{50})	[24]
60.	Amberbin A	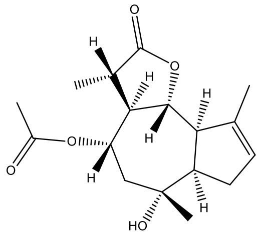	*Amberboa ramosa*	8.6 ± 0.15 μM (IC_{50})	4.8 ± 0.15 μM (IC_{50})	[24]
61.	Amberbin B	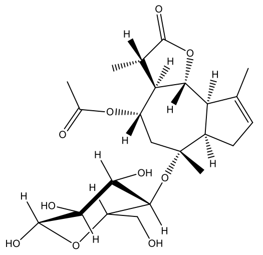	*Amberboa ramosa*	0.91 ± 0.015 μM (IC_{50})	2.5 ± 0.15 μM (IC_{50})	[24]
62.	gaillardin	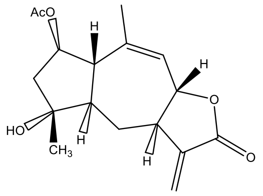	*Inula spp*	67 % (300 μg/mL)	-	[25]

63.	britannin	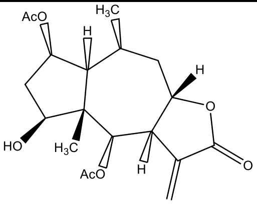	*Inula spp*	25.2 % (300 μg/mL)	-	[25]
64.	Pulchellin C	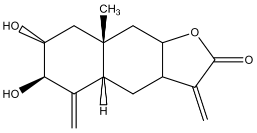	*Inula spp*	10.9 % (300 μg/mL)	-	[25]
65.	1α,6β,8α-Triacetoxy-9β-furoyloxy-β-agarofuran	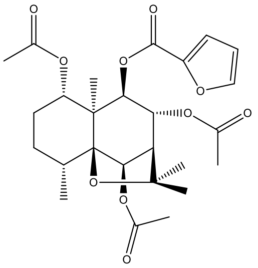	*Maytenus disticha*	0.098 ± 0.012 mg/mL (IC_{50})	>0.500 mg/mL (IC_{50})	[26]
66.	1α-Hydroxy-6β,8α-diacetoxy-9β-furoyloxy-β-agarofuran	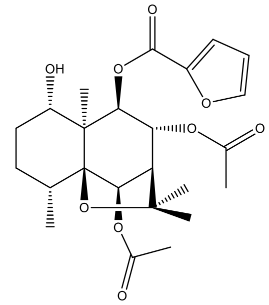	*Maytenus disticha*	0.341 ± 0.016 mg/mL (IC_{50})	>0.500 mg/mL (IC_{50})	[26]
67.	1α,6β-Diacetoxy-8α-hydroxy-9β-furoyloxy-β-agarofuran	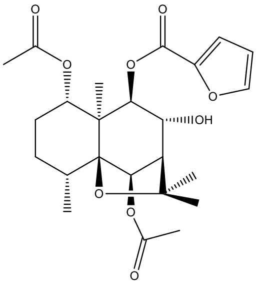	*Maytenus disticha*	0.102 ± 0.004 mg/mL (IC_{50})	>0.500 mg/mL (IC_{50})	[26]
68.	1α-Acetoxy-6β,8α-dihydroxy-9β-furoyloxy-β-agarofuran	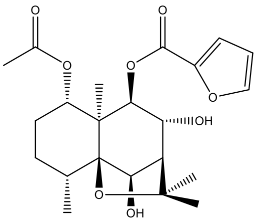	*Maytenus disticha*	0.097 ± 0.006 mg/mL (IC_{50})	>0.500 mg/mL (IC_{50})	[26]
69.	1α,2α,6β,8α,15-Pentaacetoxy-9β-benzoyloxy-β-agarofuran	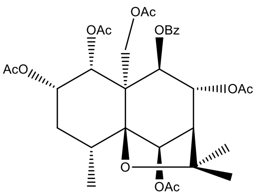	*Euonymus japonicus*	0.070 ± 0.002 mg/mL (IC_{50})	>0.500 mg/mL (IC_{50})	[26]

70.	1α,2α,3β,15-Tetraacetoxy-6β,9β-dibenzoyl-8-oxo-β-agarofuran	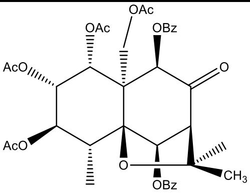	*Maytenus disticha*	0.299 ± 0.015 mg/mL (IC_{50})	>0.500 mg/mL (IC_{50})	[26]
71.	1α,6β,15-Triacetoxy-9-benzoyloxy-β-agarofuran	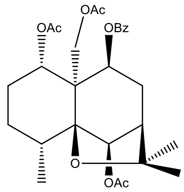	*Euonymus japonicus*	0.359 ± 0.006 mg/mL (IC_{50})	>0.500 mg/mL (IC_{50})	[26]
72.	2α,3β,6β,8α,15-Pentaacetoxy-1α,9β-benzoyloxy-β-agarofuran	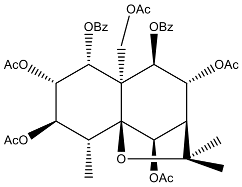	*Euonymus japonicus*	0.363 ± 0.009 mg/mL (IC_{50})	>0.500 mg/mL (IC_{50})	[26]
73.	1α-Acetoxy-6β,9β-difuroyloxy-4β-hydroxy-β-agarofuran	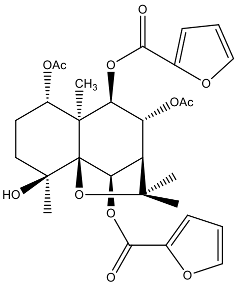	*Maytenus disticha*	0.381 ± 0.007 mg/mL (IC_{50})	>0.500 mg/mL (IC_{50})	[26]
74.	2β-Hydroxy-3α-O-caffeoyltaraxar-14-en-28-oic acid	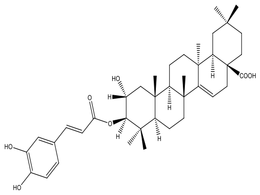	*Garcinia hombroniana*	13.5 ± 0.95 μM (IC_{50})	10.6 ± 0.54 μM (IC_{50})	[20]
75.	taraxerol	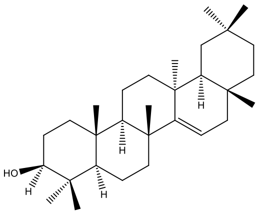	*Garcinia hombroniana*	-	17.8 ± 1.73 μM (IC_{50})	[20]
76.	Glycyrrhetinic acid	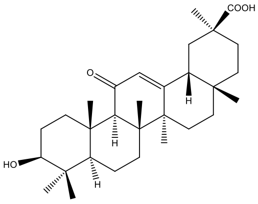	*Perovskia atriplicifolia*	54.53 ± 0.05 μM (IC_{50})	34.52 ± 0.04 μM (IC_{50})	[27]
77.	2α,3β-Dihydroxyolean-12-en-28-methyl ester	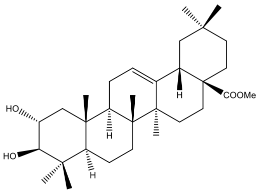	*Perovskia atriplicifolia*	46.51 ± 0.05 μM (IC_{50})	28.06 ± 0.06 μM (IC_{50})	[27]

78.	2α,3β-Dihydroxyolean-12-en-28-oic acid	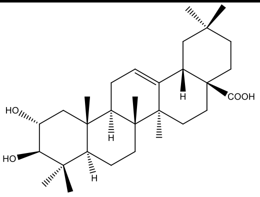	*Perovskia atriplicifolia*	33.50 ± 0.05 µM (IC_{50})	19.04 ± 0.05 µM (IC_{50})	[27]
79.	2α,3β,24-Trihydroxyolean-12-en-28-oic acid	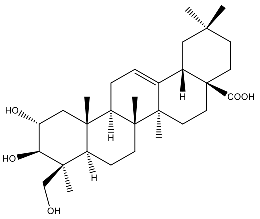	*Perovskia atriplicifolia*	24.57 ± 0.05 µM (IC_{50})	9.50 ± 0.03 µM (IC_{50})	[27]
80.	2α,3β,19β-Trihydroxyurs-12-en-28-oic acid	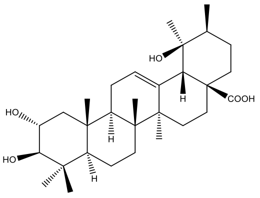	*Perovskia atriplicifolia*	29.54 ± 0.05 µM (IC_{50})	13.52 ± 0.03 µM (IC_{50})	[27]
81.	ursolic acid	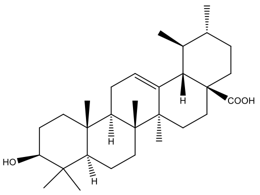	*Calceolaria talcana*	-	168.1 µg/mL (IC_{50})	[28]
82.	Loliolide	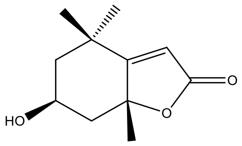	*Portulaca oleracea*	75.67 ± 0.37 µM (IC_{50})	-	[29]
83.	Isololiolide	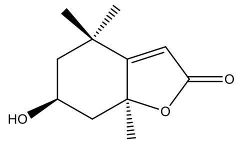	*Portulaca oleracea*	76.41 ± 0.23 µM (IC_{50})	-	[29]
84.	Dehydrololiolide	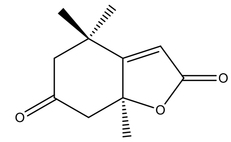	*Portulaca oleracea*	78.74 ± 0.36 µM (IC_{50})	-	[29]
85.	5,5,7,7,11,13-hexamethyl-2-(5-methylhexyl) icosahydro-1H-cyclopenta[a]chrysen-9-ol	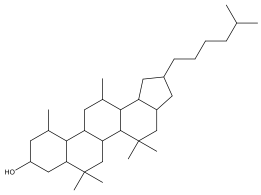	*Grewia optiva*	90 µg/mL (IC_{50})	90 µg/mL (IC_{50})	[30]
86.	Columbin	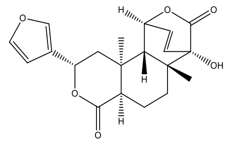	*Tinospora cordifolia*	1.2993±0.17 mg/mL (IC_{50})	-	[31]

87.	5-allosyloxy-aucubine	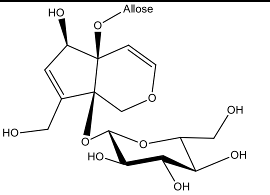	*Sideritis germanicopolitana*	20.36±0.70% (250 μg/mL)	14.22±0.61 % (250 μg/mL)	[32]
88.	Melittoside	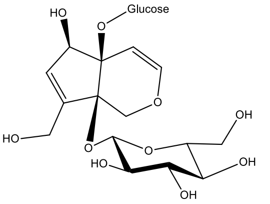	*Sideritis germanicopolitana*	24.63±0.44% (250 μg/mL)	12.75±0.40 % (250 μg/mL)	[32]
89.	Ajugol	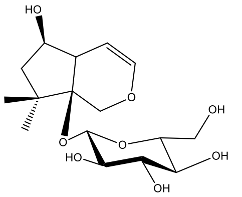	*Sideritis germanicopolitana*	21.29±0.48% (250 μg/mL)	10.51±0.32 % (250 μg/mL)	[32]
90.	Friedeline	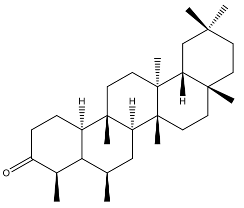	*Celtis adolphi-friderici*	-	62.3±0.21 μM (IC_{50})	[33]
91.	3-O-[2′- (2″-O-glycolyl)-glyoxylyl-β-D-glucuronopyranosyl]-28-O-β-D-glucopyranosyl-olean-12-en-3β-ol-28-oic acid	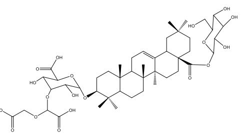	*Bassia indica*	63.1±1.5 μg/mL (IC_{50})	-	[34]
92.	(2′R,3′S)-3-O-[2′-hydroxy-3′-(2″-O-glycolyl)-oxo-propionic acid-β-D-glucuronopyranosyl]- 28-O-β-D-glucopyranosyl-olean-12-en-3β-ol-28-oic acid	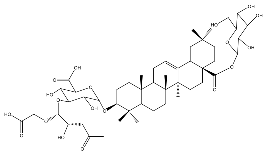	*Bassia indica*	29.6±1.7 μg/mL (IC_{50})	-	[34]
93.	(1R,15R)-1-Acetoxycryptotanshinone	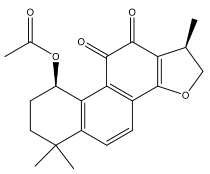	*Perovskia atriplicifolia*	22.8±2.4% (10 μg/mL)	0.84±0.09 μg/mL (IC_{50})	[35]

94.	(1R)-1-Acetoxy-tanshinone IIA	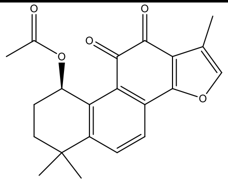	*Perovskia atriplicifolia*	28.0±0.9% (10 μg/mL)	2.77±0.48 μg/mL (IC_{50})	[35]
95.	(15R)-1-oxoaegyptinone A	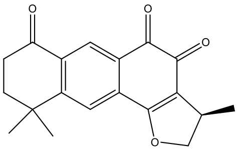	*Perovskia atriplicifolia*	49.6±1.8% (10 μg/mL)	15.75±1.12 μg/mL (IC_{50})	[35]
96.	Isograndifoliol	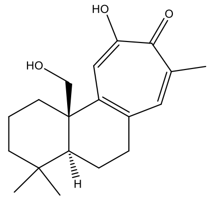	*Perovskia atriplicifolia*	50.0±1.8% (10 μg/mL)	0.27±0.02 μg/mL (IC_{50})	[35]
97.	Tunispinoside A	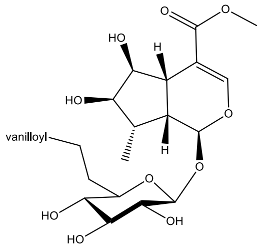	*Citharexylum spinosum*	-	30.79±1.21 μM (IC_{50})	[36]
98.	Tunispinoside B	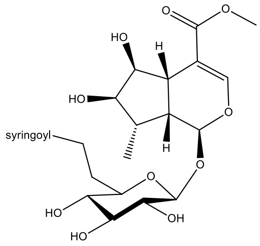	*Citharexylum spinosum*	-	17.19±1.02 μM (IC_{50})	[36]
99.	Tunispinoside C	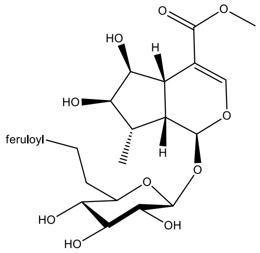	*Citharexylum spinosum*	-	52.24±2.50 μM (IC_{50})	[36]
100.	Tunispinoside D	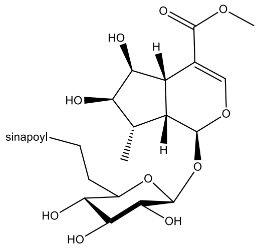	*Citharexylum spinosum*	-	35.89±1.17 μM (IC_{50})	[36]

101.	*E*-6-O-p-coumaroyl scandoside methyl ester	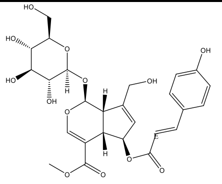	*Hedyotis diffusa*	304.18±12.15 μM (IC_{50})	98.96±2.74 μM (IC_{50})	[37]
102.	6-O-p-coumaroyl scandoside methyl ester	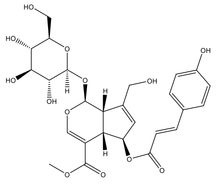	*Hedyotis diffusa*	297.84±22.68 μM (IC_{50})	26.22±1.76 μM (IC_{50})	[37]
103.	*E*-6-O-feruloyl scandoside methyl ester	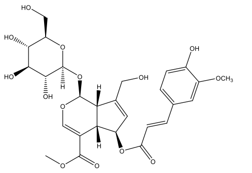	*Hedyotis diffusa*	96.84±5.29 μM (IC_{50})	116.09±29.39 μM (IC_{50})	[37]
104.	Deacetylasperulosidic acid methyl ester	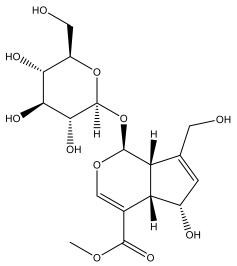	*Hedyotis diffusa*	172.26±20.55 μM (IC_{50})	17.59±0.78 μM (IC_{50})	[37]
105.	Asperuloside	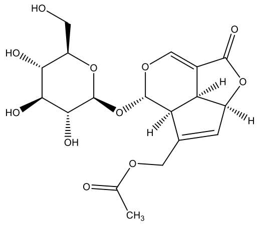	*Hedyotis diffusa*	258.81±7.48 μM (IC_{50})	>500 μM (IC_{50})	[37]
106.	6-O-Methyldeacetylasperulosidic acid methyl ester	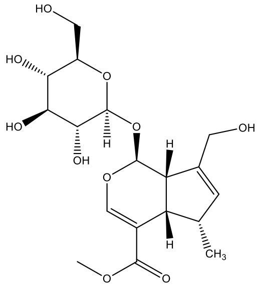	*Hedyotis diffusa*	81.06±5.58 μM (IC_{50})	32.24±2.80 μM (IC_{50})	[37]
107.	6-O-Methylscandoside methyl ester	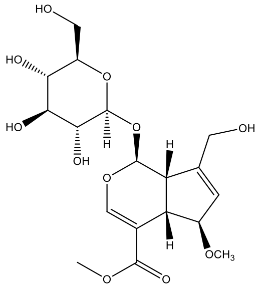	*Hedyotis diffusa*	-	11.59±0.68 μM (IC_{50})	[37]

108.	scandoside methyl ester	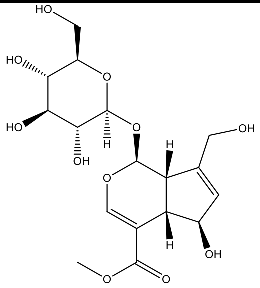	*Hedyotis diffusa*	157.68±13.18 μM (IC_{50})	16.18±2.05 μM (IC_{50})	[37]
109.	asperulosidic acid	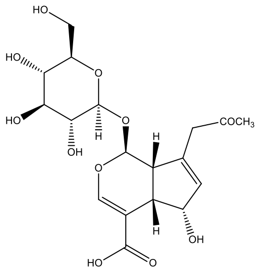	*Hedyotis diffusa*	68.34±5.11 μM (IC_{50})	80.29±19.76 μM (IC_{50})	[37]
110.	Rhotomentodione D	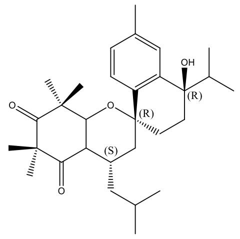	*Rhodomyrtus tomentosa*	22.9±1.1 μM (IC_{50})	-	[38]
111.	Paecilacadinol A	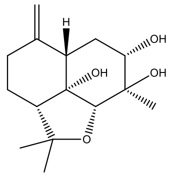	*Paecilomyces sp. TE-540*	27.05±3.21% (40 μM)	-	[39]
112.	Paecilacadinol B	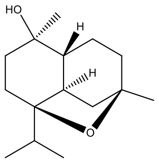	*Paecilomyces sp. TE-540*	34.23±1.15% (40 μM)	-	[39]
113.	Ustusol D	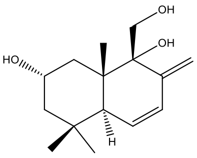	*Paecilomyces sp. TE-540*	27.29±2.07% (40 μM)	-	[39]
114.	Ustusol E	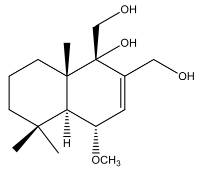	*Paecilomyces sp. TE-540*	41.35±0.65% (40 μM)	-	[39]
115.	12-hydroxyalbrassitriol	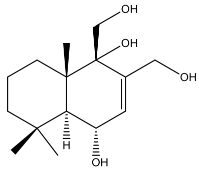	*Paecilomyces sp. TE-540.*	43.02±6.01 μM (IC_{50})	-	[39]

116.	2-hydroxyalbrassit-riol	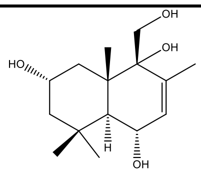	*Paecilomyces sp. TE-540*	35.97±2.12 μM (IC_{50})	-	[39]
117.	Deoxyuvidin B	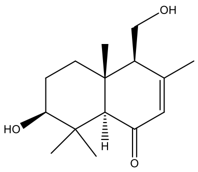	*Paecilomyces sp. TE-540*	19.23±1.89% (40 μM)	-	[39]
118.	3β,9α,11-trihydroxy-6-oxodrim-7-ene	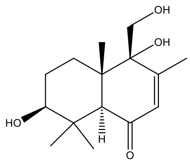	*Paecilomyces sp. TE-540*	33.04±4.30% (40 μM)	-	[39]
119.	2α,11-dihydroxy-6-oxodrim-7-ene	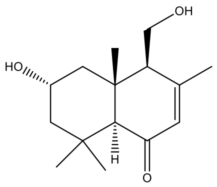	*Paecilomyces sp. TE-540sp.*	17.56±3.33% (40 μM)	-	[39]
120.	Ustusol B	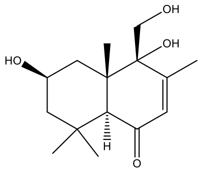	*Paecilomyces sp. TE-540*	24.24±1.35% (40 μM)	-	[39]
121.	kadcoccilactone S	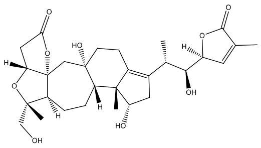	*Kadsura coccinea*	85.91±1.90 μM (IC_{50})	-	[40]
122.	micrandilactone C	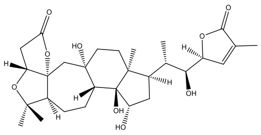	*Kadsura coccinea*	97.90±1.50 μM (IC_{50})	-	[40]
123.	micrandiactone H	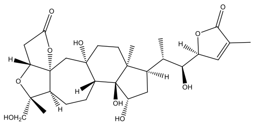	*Kadsura coccinea*	86.99±1.04 μM (IC_{50})	-	[40]
124.	seco-coccinic acid A	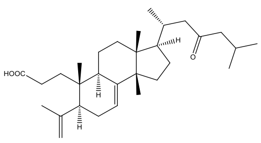	*Kadsura coccinea*	91.14±2.73 μM (IC_{50})	-	[40]
125.	seco-coccinic acid G	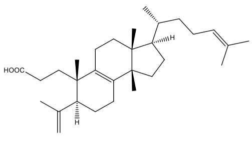	*Kadsura coccinea*	73.16±1.78 μM (IC_{50})	-	[40]

126.	sandaracopimaric acid	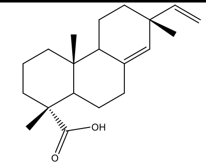	*Cupressus macrocarpa*	195.99 μg/mL (IC_{50})	-	[41]
127.	Agathadiol	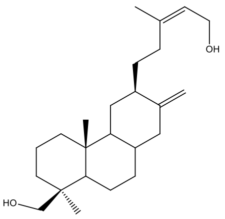	*Cupressus macrocarpa*	228.47 μg/mL (IC_{50})	-	[41]
128.	3-O-detigloyl-3-O-isobutyrylfebrifugin A	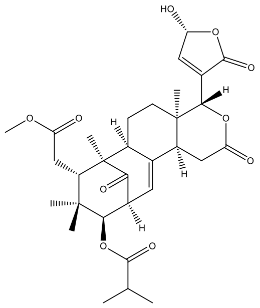	*Cipadessa baccifera*	25.69±1.84% (50 μM)	-	[42]
129.	Granatumin E	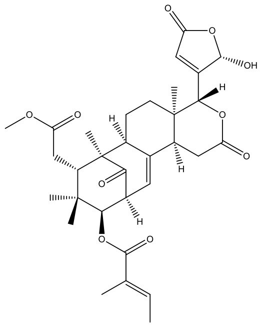	*Cipadessa baccifera*	23.98±1.55% (50 μM)	-	[42]
130.	Khaysin T	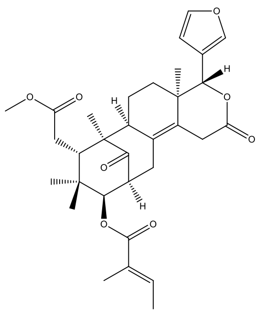	*Cipadessa baccifera*	25.13±3.55% (50 μM)	-	[42]
131.	2′S-cipadesin A	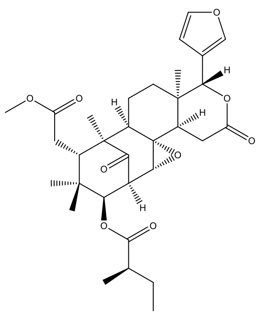	*Cipadessa baccifera*	38.76±4.33% (50 μM)	-	[42]

Table S 2: Phenolic compounds as Acetyl and Butyryl cholinesterase inhibitors

Number	Name	Structure	Plant source	IC_{50} or %inh (conc.)		Reference
				**AChE**	**BChE**	
1.	Quercetin	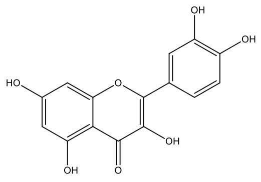	*Aristotelia chilensis*	47.8 μg/mL (IC_{50})	38.8 μg/mL (IC_{50})	[43]
			Eugenia dysenterica	46.59±0.49 μg/mL (IC_{50})	-	[44]
			Leiotulus dasyanthus	-	6.98 ± 3.01 % (20 μg/mL)	[45]
			Cleistocalyx perculatus	25.9 ± 1.7 μM (IC_{50})	177.8 ± 11.9 μM (IC_{50})	[46]
			Bassia indica	18±1.3 μg/mL (IC_{50})	-	[34]
2.	Myricetin	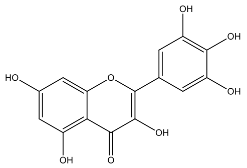	*Aristotelia chilensis*	37.2 μg/mL (IC_{50})	70.7 μg/mL (IC_{50})	[43]
3.	Rhamnetin	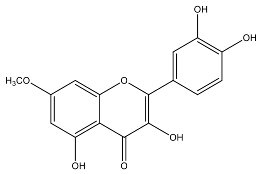	*Aristotelia chilensis*	89.9 μg/mL (IC_{50})	7.8 μg/mL (IC_{50})	[43]
4.	Apigenin	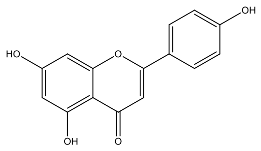	*Aristotelia chilensis*	19.7 μg/mL (IC_{50})	24.5 μg/mL (IC_{50})	[43]
			Thunbergia erecta	37.33 ± 4.02 ng/mL (IC_{50})	-	[47]
			Helichrysum plicatum	1.78 μM (IC_{50})	1.88 μM (IC_{50})	[48]
5.	Luteolin	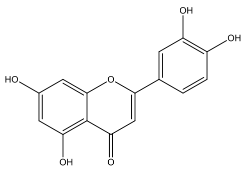	*Aristotelia chilensis*	15.9 μg/mL (IC_{50})	49.8 μg/mL (IC_{50})	[43]
			Achillea millefolium	2.12 ± 0.145 μM (IC_{50})	2.45 ± 0.099 μM (IC_{50})	[49]

6.	Tamarixetin	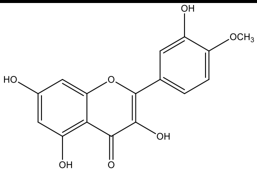	*Cleistocalyx perculatus*	22.3 ± 2.2 μM (IC_{50})	160.6 ± 3.8 μM (IC_{50})	[46]
7.	Kaempferol	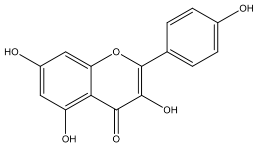	*Cleistocalyx perculatus*	30.4 ± 2.2 μM (IC_{50})	62.5 ± 1.3 μM (IC_{50})	[46]
			Eupatorium adenophorum	24.92±0.49 μg/mL (IC_{50})	-	[50]
8.	Santin	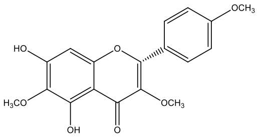	*Dodonaea viscosa*	>300 μM (IC_{50})	274.07 ± 1.68 μM (IC_{50})	[51]
9.	Penduletin	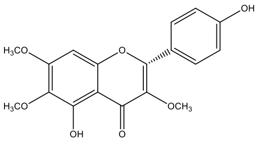	*Dodonaea viscosa*	175.11 ± 1.36 μM (IC_{50})	55.78 ± 1.01 μM (IC_{50})	[51]
10.	Viscosine	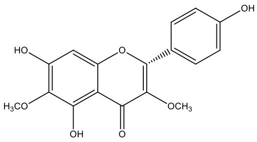	*Dodonaea viscosa*	182.97 ± 1.25 μM (IC_{50})	47.07 ± 0.54 μM (IC_{50})	[51]
11.	6,7-dimethylkaempferol	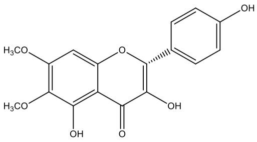	*Dodonaea viscosa*	>300 μM (IC_{50})	145.65 ± 1.45 μM (IC_{50})	[51]
12.	Kaempferol-3- methylether	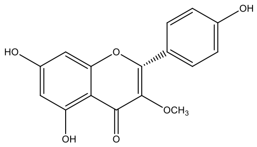	*Dodonaea viscosa*	270.25 ± 1.16 μM	115.97 ± 1.18 μM	[51]
13.	3,4′-dimethoxy-5,7-dihydroxyflavone	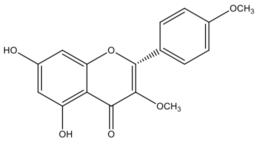	*Dodonaea viscosa*	>300 μM (IC_{50})	298.40 ± 2.01 μM (IC_{50})	[51]
14.	Kaempferide	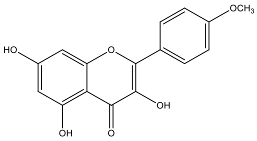	*Alpinia officinarum*	31.9 ± 2.0 μM (IC_{50})	47.6 ± 1.6 (100 μM) (IC_{50})	[52]
15.	Galangin	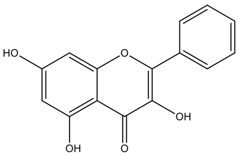	*Alpinia officinarum*	70.1 ± 1.5 μM (IC_{50})	61.4 ± 1.4 μM (IC_{50})	[52]

16.	Acacetin	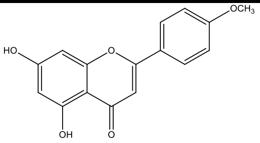	*Aristotelia chilensis*	112.3 μg/mL (IC_{50})	177.8 μg/mL (IC_{50})	[43]
17.	Diosmetin	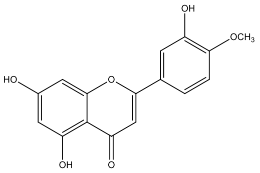	*Aristotelia chilensis*	45.6 μg/mL (IC_{50})	12.9 μg/mL (IC_{50})	[43]
18.	Cirsilineol	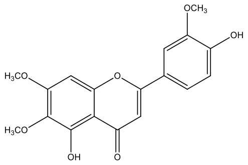	*Ocimum sanctum*	2.95 ± 0.02 μM (IC_{50})	3.25 ± 0.08 μM (IC_{50})	[53]
19.	Isothymusin	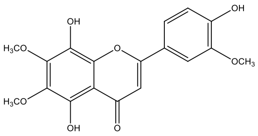	*Ocimum sanctum*	8.25 ± 0.13 μM (IC_{50})	7.85 ± 0.01 μM (IC_{50})	[53]
20.	Myricetin-3'-methylether 3-O-β-D-galactopyranoside	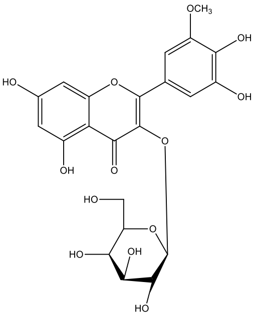	*Cleistocalyx perculatus*	19.9 ± 0.6 μM (IC_{50})	152.5 ± 0.4 μM (IC_{50})	[46]
21.	Myricetin-3',5'-di-methylether 3-O-β-D-galactopyranoside	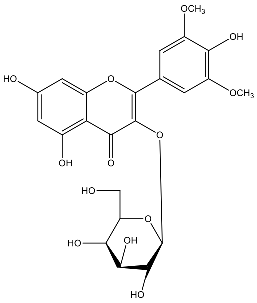	*Cleistocalyx perculatus*	37.8 ± 1.0 μM (IC_{50})	> 800 μM (IC_{50})	[46]
22.	Quercitrin	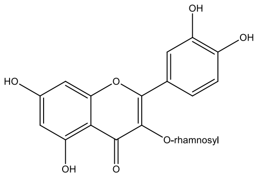	*Aristotelia chilensis*	66.9 μg/mL (IC_{50})	78.8 μg/mL (IC_{50})	[43]
23.	Rutin	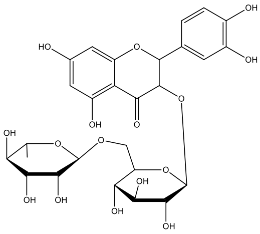	*Aristotelia chilensis*	169.8 μg/mL (IC_{50})	95.1 μg/mL (IC_{50})	[43]
			Leiotulus dasyanthus	-	5.95 ± 1.09 % (20 μg/mL)	[45]

			Eupatorium adenophorum	66.97±0.15 µg/mL (IC_{50})	-	[50]
24.	Isoorientin-6-O″-β-D-glucopyranoside	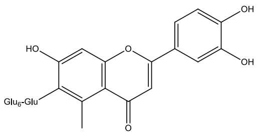	*Iris pseudopumila*	60.8±1.8 µM (IC_{50})	98.9±1.4 µM (IC_{50})	[54]
25.	Isovitexin-6-O″-β-D-glucopyranoside	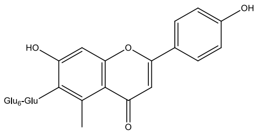	*Iris pseudopumila*	85.9±3.6 µM (IC_{50})	>100 µM (IC_{50})	[54]
26.	kaempferol-3-O-β-D-glucopyranoside	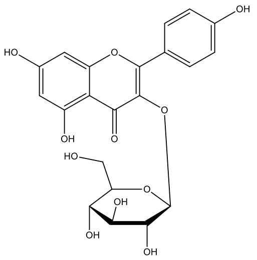	*Iris pseudopumila*	50.4±1.4 µM (IC_{50})	76.8±1.7 µM (IC_{50})	[54]
			Eupatorium adenophorum	61.44±1.81 µg/mL (IC_{50})	-	[50]
27.	Apigenin-7-O-β-D-glucoside	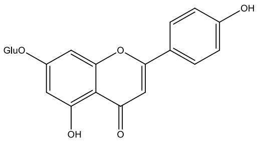	*Achillea millefolium*	1.89 ± 0.067 µM (IC_{50})	2.13 ± 0.440 µM (IC_{50})	[49]
28.	Luteolin 7-O-β-D-glucoside	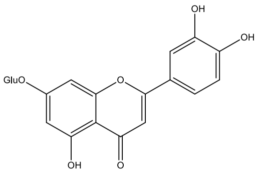	*Achillea millefolium*	1.67 ± 0.302 µM (IC_{50})	2.20 ± 0.058 µM (IC_{50})	[49]
29.	6-OH-luteolin 7-O-β-D-glucoside	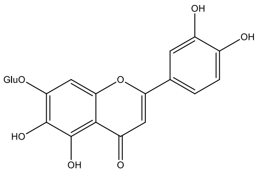	*Achillea millefolium*	1.65 ± 0.122 µM (IC_{50})	1.97 ± 0.220 µM (IC_{50})	[49]
30.	Isoorientin	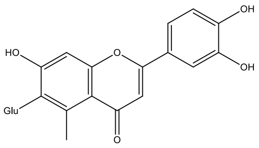	*Iris pseudopumila*	26.8±0.8 µM (IC_{50})	31.5±0.7 µM (IC_{50})	[54]
31.	Isovitexin	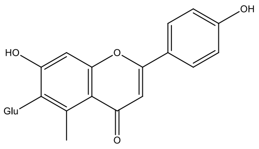	*Iris pseudopumila*	36.4±2.1 µM (IC_{50})	54.8±2.5 µM (IC_{50})	[54]

32.	kaempferol 3-O-(3′′-O-E-p-coumaroyl)-(6′′-O-E-feruloyl)-β-glucopyranoside	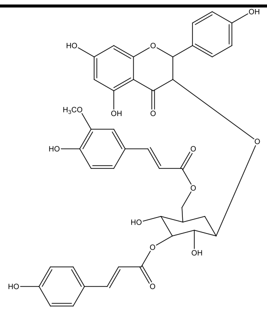	*Stenochlaena palustris*	-	113.66 ± 7.66 μM (IC_{50})	[55]
33.	kaempferol 3-O-(3′′,6′′-di-O-E-p-coumaroyl)-β-glucopyranoside	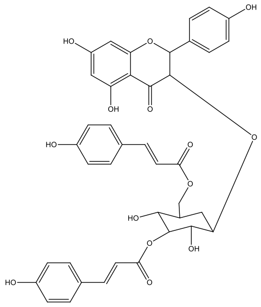	*Stenochlaena palustris*	-	85.37 ± 7.62 μM (IC_{50})	[55]
34.	8-prenylnaringenin	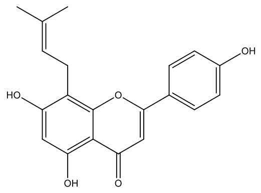	*Humulus lupulus*	28.15 ± 1.38 % (100 μM)	54.05 ± 2.89 % (100 μM)	[56]
35.	5,7,4′-trihydroxy-3′-(3-hydroxymethylbutyl)-3,6-dimethoxyflavone	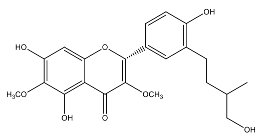	*Dodonaea viscosa*	>300 μM (IC_{50})	222.54 ± 1.64 μM (IC_{50})	[51]
36.	5,7-dihydroxy-3′-(2-hydroxy-3-methyl-3-butenyl)-3,6,4′-trimethoxyflavone	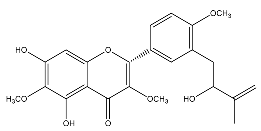	*Dodonaea viscosa*	>300 μM (IC_{50})	>300 μM (IC_{50})	[51]
37.	5,7-dihydroxy-3′-(3-hydroxymethylbutyl)-3,6,4′-trimethoxyflavone	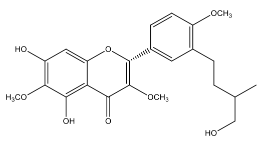	*Dodonaea viscosa*	>300 μM (IC_{50})	192.60 ± 1.96 μM (IC_{50})	[51]

38.	5,7-dihydroxy-3′-(4′′-acetoxy-3′′-methyl-butyl)-3,6,4′-trimethoxy flavone	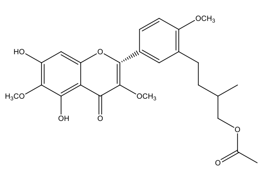	*Dodonaea viscosa*	>300 μM (IC_{50})	>300 μM (IC_{50})	[51]
39.	Triacetyl tricin	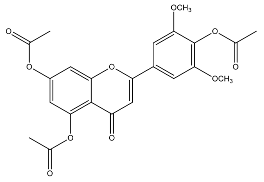	*Rhynchospora corymbosa*	-	67.36 ± 0.84 μM (IC_{50})	[21]
40.	Diacetyl tricin	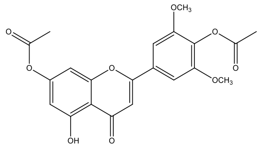	*Rhynchospora corymbosa*	-	58.91 ± 0.43 μM (IC_{50})	[21]
41.	Monoacetyl tricin	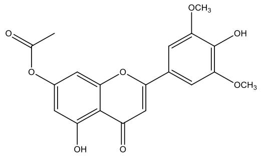	*Rhynchospora corymbosa*	-	24.25 ± 0.21 μM (IC_{50})	[21]
42.	p-Hydroxybenzoic acid	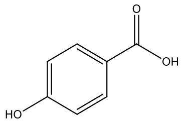	*Nelumbo nucifera*	20.07±0.07 % (10 μg/mL)	62.29±1.18 % (10 μg/mL)	[17]
43.	Gallic acid	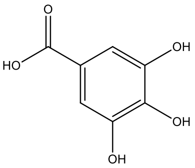	*Orostachys japonicus*	185.2 ± 2.1 μM (IC_{50})	1000 μM (IC_{50})	[57]
44.	Ferulic acid methyl ester	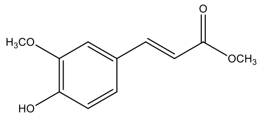	*Cimicifuga dahurica*	58.0 ± 0.6 % (100 μM)	62.3 ± 0.3 % (100 μM)	[18]
45.	methyl gallate	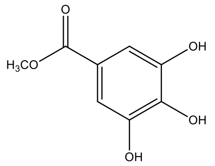	*Orostachys japonicus*	171.2 ± 1.5 μM (IC_{50})	1000 μM (IC_{50})	[57]
46.	vanilloloside	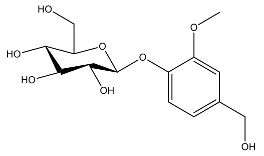	*Nelumbo nucifera*	4.55±0.72 % (10 μg/mL)	205.78±0.79 % (10 μg/mL)	[17]
47.	4-*O*-caffeoyl-quinic acid	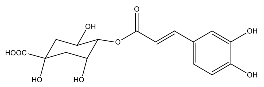	*Acanthopanax henryi*	80.2 ± 2.2 μM (IC_{50})	-	[58]

48.	4,5-di-caffeoylquinic acid	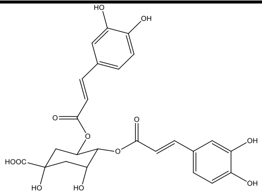	*Acanthopanax henryi*	62.6 ± 8.3 μM (IC_{50})	-	[58]
49.	Piceatannol	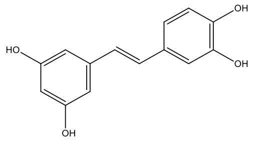	*Belamcandae chinensis rhizoma*	67.07 ± 1.52 % (100 μg/mL)	91.10 ± 1.26 % (100 μg/mL)	[59]
50.	Resveratrol	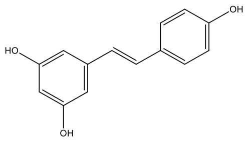	*Belamcandae chinensis rhizoma*	14.43 ± 1.46 % (100 μg/mL)	56.30 ± 2.33 % (100 μg/mL)	[59]
			Rheum lhasaense	1709±56.56 μM (IC_{50})	-	[60]
51.	Cimiciphenone	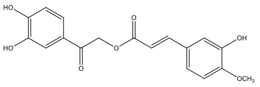	*Cimicifuga dahurica*	65.4 ± 0.9 % (100 μM)	13.6 ± 0.7 % (100 μM)	[18]
52.	Rosmarinic acid	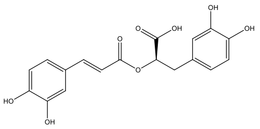	*Perovskia atriplicifolia*	28.18 ± 5.13 % (10 μg/mL)	80.74 ± 4.03 % (10 μg/mL)	[16]
53.			*Thunbergia erecta*	83.02 ± 4.74 ng/mL (IC_{50})	-	[47]
54.	(4E)-1,7-diphenyl-4-hepten-3-one	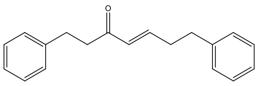	*Alpinia officinarum*	23.9 ± 2.6 μM (IC_{50})	70.7 ± 2.5 μM (IC_{50})	[52]
55.	(4E)-7-(4-hydroxyphenyl)- 1-phenyl-4-hepten-3-one	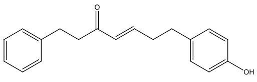	*Alpinia officinarum*	87.3 ± 3.4 μM (IC_{50})	41.1 ± 0.1 % (100 μM)	[52]
56.	(4E)-7-(4-hydroxy-3-methoxyphenyl)-1-phenyl-hept-4-en-3-one	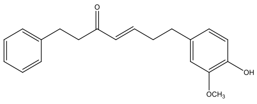	*Alpinia officinarum*	39.1 ± 2.3 μM (IC_{50})	43.7 ± 1.4 % (100 μM)	[52]
57.	Dihydroyashsbushiketol	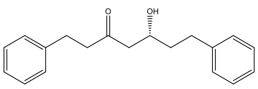	*Alpinia officinarum*	36.2 ± 1.9 % (100 μM)	15.7 ± 2.1 % (100 μM)	[52]
58.	(5R)-7-(4-hydroxy-3-methoxyphenyl)-5-methoxy-1-	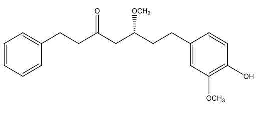	*Alpinia officinarum*	35.3 ± 1.0 % (100 μM)	21.5 ± 0.6 % (100 μM)	[52]

	phenyl-3-heptanone					
59.	malabaricone E	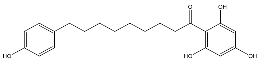	*Myristica cinnamomea*	6.44 ± 0.85 μM (IC_{50})	6.65 ± 0.13 μM (IC_{50})	[61]
60.	Malabaricones A	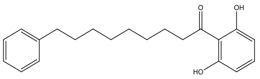	*Myristica cinnamomea*	1.31 ± 0.17 μM (IC_{50})	39.21 ± 3.46 μM (IC_{50})	[61]
61.	Malabaricones B	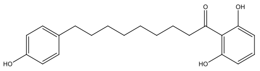	*Myristica cinnamomea*	1.84 ± 0.19 μM (IC_{50})	1.76 ± 0.21 μM (IC_{50})	[61]
62.	Malabaricone C	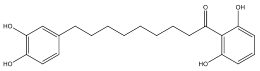	*Myristica cinnamomea*	1.94 ± 0.27 μM (IC_{50})	2.80 ± 0.49 μM (IC_{50})	[61]
			Myristica fragrans	2.06±0.04 μg/mL (IC_{50})	-	[62]
63.	Maingayones A	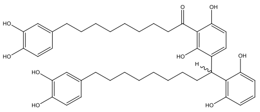	*Myristica cinnamomea*	12.66 ± 1.48 μM (IC_{50})	10.51 ± 2.07 μM (IC_{50})	[61]
64.	Maingayones B	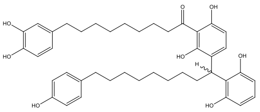	*Myristica cinnamomea*	30.67 ± 8.14 μM (IC_{50})	12.52 ± 2.86 μM (IC_{50})	[61]
65.	(-)-alpininoid B	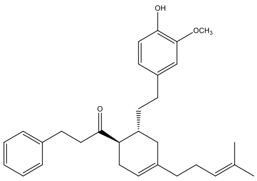	*Alpinia officinarum*	2.6 ± 4.2 μM (IC_{50})	35.2± 0.7 μM (IC_{50})	[52]
66.	Tectorigenin	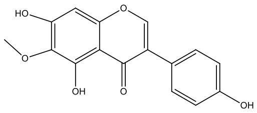	*Belamcandae chinensis rhizoma*	-	18.08 ± 2.93 % (100 μg/mL)	[59]
67.	Iristectorigenin B	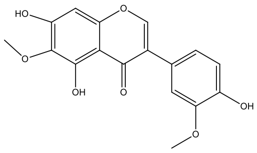	*Belamcandae chinensis rhizoma*	-	37.07 ± 0.47 % (100 μg/mL)	[59]
68.	Irigenin	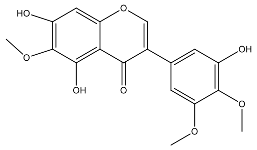	*Belamcandae chinensis rhizoma*	-	20.12 ± 2.47 % (100 μg/mL)	[59]
69.	Irilin B	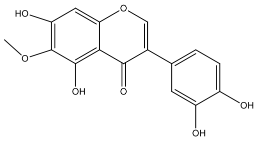	*Belamcandae chinensis rhizoma*	3.67 ± 1.44 % (100 μg/mL)	49.72 ± 2.83 % (100 μg/mL)	[59]
70.	Iridin	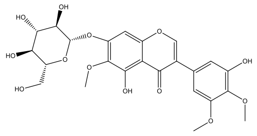	*Belamcandae chinensis rhizoma*	-	4.29 ± 2.04 % (100 μg/mL)	[59]

71.	Iristectorin B	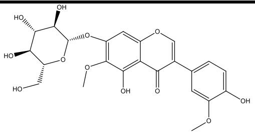	*Belamcandae chinensis rhizoma*	-	9.44 ± 0.09 % (100 μg/mL)	[59]
72.	Irisflorentin	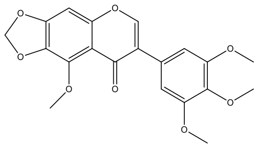	*Belamcandae chinensis rhizoma*	36.25 ± 1.22 % (100 μg/mL)	9.34 ± 2.21 % (100 μg/mL)	[59]
73.	Irilone	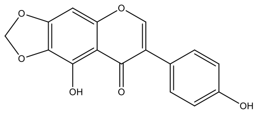	*Iris pseudopumila*	93.6±2.4 μM (IC_{50})	>100 μM (IC_{50})	[54]
74.	Irigenin-7-O-β-D-glucopyranoside	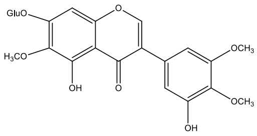	*Iris pseudopumila*	94.1±2.5 μM (IC_{50})	>100 μM (IC_{50})	[54]
75.	Irisolone-4′-O-β-D-glucopyranoside	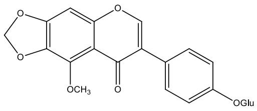	*Iris pseudopumila*	93.2±2.6 μM (IC_{50})	>100 μM (IC_{50})	[54]
76.	Osajin	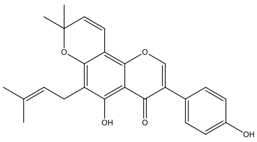	*Maclura pomifera*	2.239 mM (IC_{50})	-	[63]
77.	Pomiferin	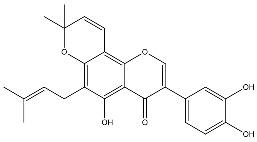	*Maclura pomifera*	0.096 mM (IC_{50})	-	[63]
78.	Iso-osajin	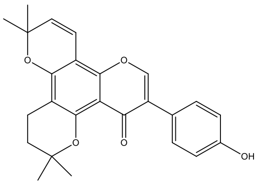	*Maclura pomifera*	1.35 mM (IC_{50})	-	[63]
79.	Iso-pomiferin	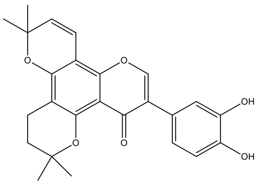	*Maclura pomifera*	2.67 mM (IC_{50})	-	[63]
80.	Catechin	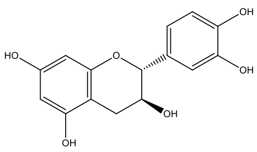	*Eugenia dysenterica*	42.39±0.67 μg/mL (IC_{50})	-	[44]
			Kadsura coccinea	7.58±2.45 μM (IC_{50})	-	[40]
81.	(+)-catechin	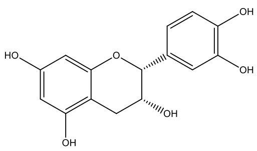	*Orostachys japonicus*	191.2 ± 1.3 μM (IC_{50})	727.3 ± 2.1 μM (IC_{50})	[57]

82.	(-)-epicate-chin-3-Ogal-late	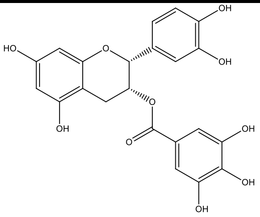	*Orostachys japonicus*	328.1 ± 2.1 μM (IC_{50})	412.5 ± 1.2 μM (IC_{50})	[57]
83.	(+)-catechin-3-O-gallate	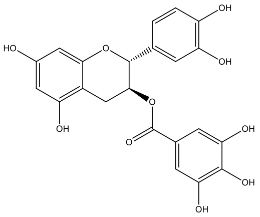	*Orostachys japonicus*	142.8 ± 3.7 μM (IC_{50})	367.3 ± 3.2 μM (IC_{50})	[57]
84.	(-)-epicate-chin-3.5-O-digallate	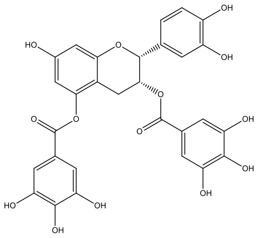	*Orostachys japonicus*	98.3 ± 2.1 μM (IC_{50})	30.4 ± 2.4 μM (IC_{50})	[57]
85.	Hesperetin	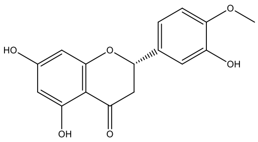	*Citrus spp.*	45.70 ± 2.69 μM (IC_{50})	>100 μM (IC_{50})	[64]
86.	Naringenin	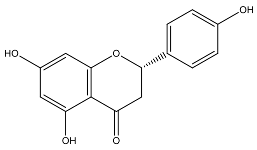	*Citrus spp.*	42.66 ± 4.30 μM (IC_{50})	>100 μM (IC_{50})	[64]
			Eupatorium adenophorum	45.23±3.74 μg/mL (IC_{50})	-	[50]
87.	Aromadendrin (2S,3S) 3,4′,5,7-tetrahydroxyflavanone	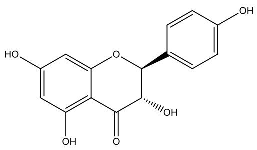	*Dodonaea viscosa*	173.22 ± 1.07 μM (IC_{50})	95.13 ± 1.24 μM (IC_{50})	[51]
88.	Pinocembrin	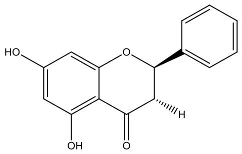	*Dodonaea viscosa*	>200 μM (IC_{50})	99.36 ± 0.87 μM (IC_{50})	[51]
89.	isoxanthohumol	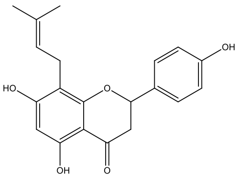	*Humulus lupulus*	-	13.87 ± 0.95 % (100 μM)	[56]
90.	6-prenylnaringenin	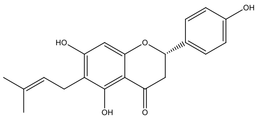	*Humulus lupulus*	8.07 ± 2.38 % (100 μM)	31.11 ± 3.92 % (100 μM)	[56]

91.	Hesperidin	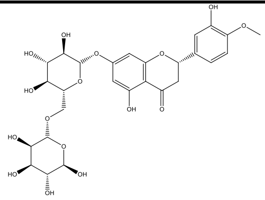	*Citrus spp*	22.80 ± 2.78 μM (IC_{50})	48.09 ± 0.74 μM (IC_{50})	[64]
92.	Nostotrebin 6	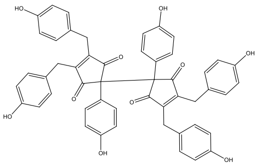	*Nostoc sp*	5.5 μM (IC_{50})	6.1 μM (IC_{50})	[65]
93.	2,6-di-methoxyl-p-benzoquinone	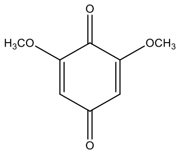	*Xylia xylocarpa*	54.4 ± 3.4 μM (IC_{50})	42.7 ± 7.6 μM (IC_{50})	[19]
94.	2-hydroxy-3-(1,1-dimethylallyl-1,4-naphthoquinone)	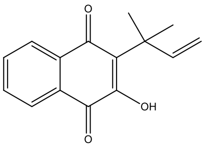	*Calceolaria talcana*	-	142.4 μg/mL (IC_{50})	[28]
95.	chrysophanol	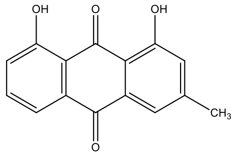	*Xylia xylocarpa*	77.3 ± 0.8 μM (IC_{50})	>100 μM (IC_{50})	[19]
96.	8-Deoxygartanin	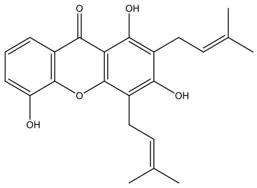	*Garcinia mangostana*	20.41 μM (IC_{50})	6.47 μM (IC_{50})	[66]
97.	Garcinone C	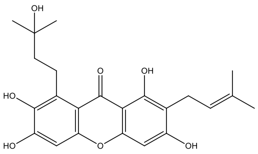	*Garcinia mangostana*	1.24 μM (IC_{50})	8.96 μM (IC_{50})	[66]
98.	Mangostanol	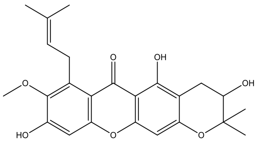	*Garcinia mangostana*	5.77 μM (IC_{50})	10.41 μM (IC_{50})	[66]
99.	3-Isomangostin	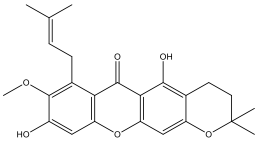	*Garcinia mangostana*	5.75 μM (IC_{50})	12.96 μM (IC_{50})	[66]
100.	α-Mangostin		*Garcinia mangostana*	2.14 μM (IC_{50})	5.41 μM (IC_{50})	[66]

		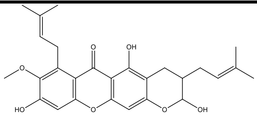	*Garcinia fusca*	2.38±0.20 μM (IC_{50})	3.18±0.05 μM (IC_{50})	[67]
101.	γ-Mangostin	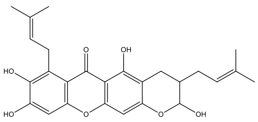	*Garcinia mangostana*	1.31 μM (IC_{50})	1.78 μM (IC_{50})	[66]
			Garcinia fusca	2.62±0.06 μM (IC_{50})	1.05±0.02 μM (IC_{50})	[67]
102.	Mangiferin	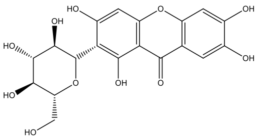	*Belamcandae chinensis rhizoma*	-	8.99 ± 2.26 % (100 μg/mL)	[59]
103.	3-hydroxyxanthohumol	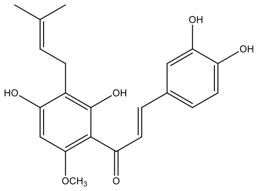	*Humulus lupulus*	72.12 ± 1.54 % (100 μM)	68.95 ± 2.54 % (100 μM)	[56]
104.	Xanthohumol	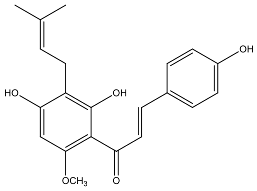	*Humulus lupulus*	55.71 ± 2.63 % (100 μM)	75.76 ± 3.77 % (100 μM)	[56]
105.	xanthohumol B	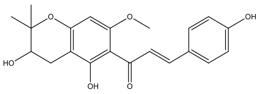	*Humulus lupulus*	18.00 ± 3.30 % (100 μM)	47.52 ± 2.02 % (100 μM)	[56]
106.	xanthohumol D	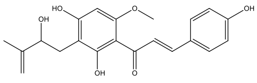	*Humulus lupulus*	9.42 ± 2.06 % (100 μM)	20.86 ± 3.61 % (100 μM)	[56]
107.	Moracin M	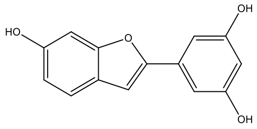	*Morus alba*	49.53 ± 1.01 μM (IC_{50})	38.08 ± 0.57 μM (IC_{50})	[68]
108.	Moracin P	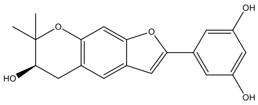	*Morus alba*	52.59 ± 1.06 μM (IC_{50})	37.96 ± 0.91 μM (IC_{50})	[68]
109.	Moracin O	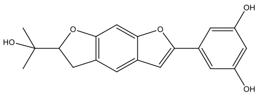	*Morus alba*	21.63 ± 3.26 μM (IC_{50})	28.22 ± 0.31 μM (IC_{50})	[68]
110.	Moracin S	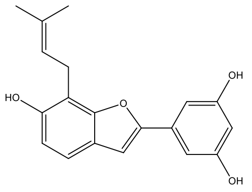	*Morus alba*	32.36 ± 0.49 μM (IC_{50})	7.22 ± 0.22 μM (IC_{50})	[68]

111.	1,2,3,6-Tetra-O-galloyl-b-D-glucose	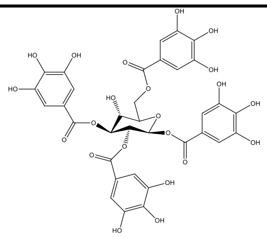	*Cornus officinalis*	85.64 ± 0.03 μg/mL (IC_{50})	70.22 ± 0.44 μg/mL (IC_{50})	[69]
112.	Tellima-grandin I	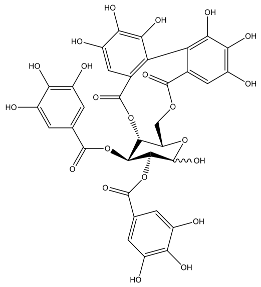	*Cornus officinalis*	87.52 ± 0.32 μg/mL (IC_{50})	92.08 ± 3.39 μg/mL (IC_{50})	[69]
113.	Tellima-grandin II	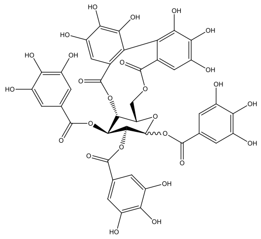	*Cornus officinalis*	11.86 ± 0.56 μg/mL (IC_{50})	18.29 ± 0.01 μg/mL (IC_{50})	[69]
114.	Isoterchebin	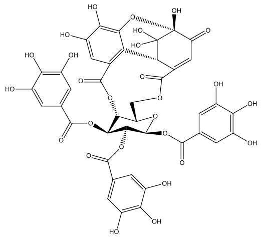	*Cornus officinalis*	47.55 ± 0.54 μg/mL (IC_{50})	20.65 ± 0.29 μg/mL (IC_{50})	[69]
115.	Isocorilagin	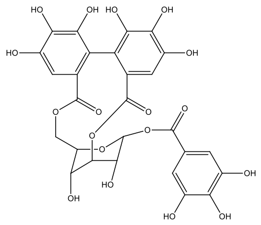	*Phyllanthus niruri*	0.49 μM (IC_{50})	4.20 μM (IC_{50})	[70]
116.	verbascoside	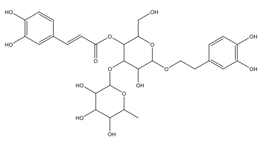	*Calceolaria talcana*	189.8 μg/mL (IC_{50})	105.9 μg/mL (IC_{50})	[28]
			Sideritis germanicopolitana	25.18±0.62% (250 μg/mL)	13.11±0.51% (250 μg/mL)	[32]
			Citharexylum spinosum	-	80.24±2.15 μM (IC_{50})	[36]

117.	Garcinefla-vanone A	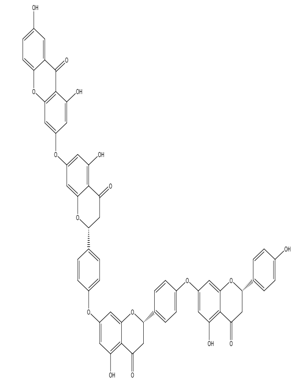	*Garcinia atroviridis*	80.15 ± 6.65 % (100 μM)	12 ± 3.84 % (100 μM)	[71]
118.	Garcineflavo-nol A	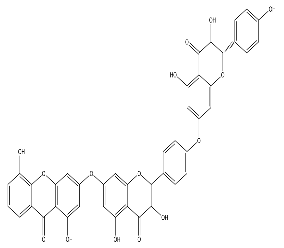	*Garcinia atroviridis*	68.45 ± 0.97 % (100 μM)	54.66 ± 3.87 % (100 μM)	[71]
119.	Oleralignan A	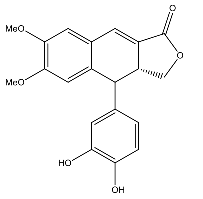	*Portulaca oleracea*	58.31 ± 0.23 μM (IC_{50})	-	[29]
120.	trans-couma-ric acid me-thyl ester	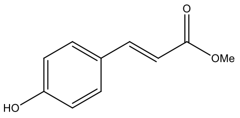	*Portulaca oleracea*	93.88 ± 0.39 μM (IC_{50})	-	[29]
121.	Vanillic acid	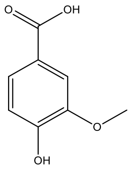	*Thunbergia erecta*	30.8 ± 1.61 ng/mL (IC_{50})	-	[47]
			Croton oligandrus	-	62.2±0.06 μM (IC_{50})	[72]
			Bassia indica	88.3±1.10 μg/mL (IC_{50})	-	[34]
122.	trans-Ferulic acid	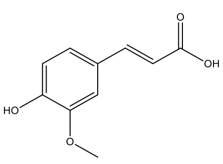	*Thunbergia erecta*	127.9 ± 7.31 ng/mL (IC_{50})	-	[47]
123.	3,4,5-Tri-methoxyphe-nol-1-O-β-D-glucoside	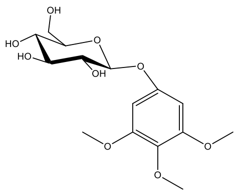	*Thunbergia erecta*	212.03 ± 9.31 ng/mL (IC_{50})	-	[47]
124.	Acacetin-7-O-β-D-gluco-side	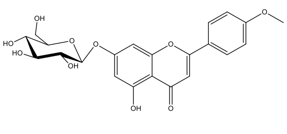	*Thunbergia erecta*	49.57 ± 2.14 ng/mL (IC_{50})	-	[47]

125.	Acacetin 7-O-(α-D-apio-furanosyl) (1 → 6)-β-D-glucoside	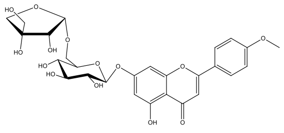	*Thunbergia erecta*	372.70 ± 21.3 ng/mL (IC_{50})	-	[47]
126.	Benzyl-7-O-β-xylopyranosyl (1″→ 2′)-β-D-glucoside	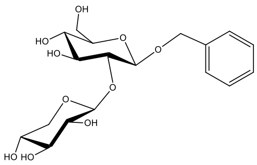	*Thunbergia erecta*	107.70 ± 6.16 ng/mL (IC_{50})	-	[47]
127.	p-Coumaric acid	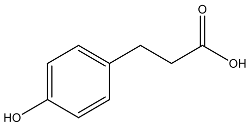	*Ziziphus oxyphylla*	80 μg/mL (IC_{50})	80 μg/mL (IC_{50})	[73]
128.	3,4-di-methoxy benzoic acid	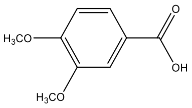	*Ziziphus oxyphylla*	90 μg/mL (IC_{50})	91 μg/mL (IC_{50})	[73]
129.	4-Heptyloxy benzoic acid	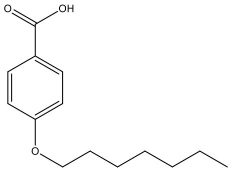	*Ziziphus oxyphylla*	89 μg/mL (IC_{50})	90 μg/mL (IC_{50})	[73]
130.	Tomentocarpan A	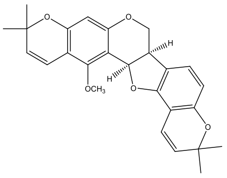	*Lespedeza tomentosa*	-	0.50 mM (IC_{50})	[74]
131.	Tomentocarpan B	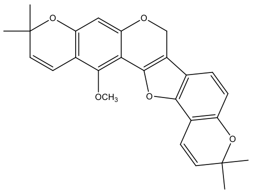	*Lespedeza tomentosa*	-	0.62 mM (IC_{50})	[74]
132.	Pholidotaphenanthrenequinone A	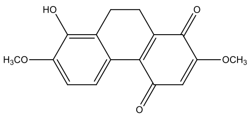	*Pholidota cantonensis*	59.96±1.88% (50 μg/mL)	-	[75]
133.	2,7-Dihydroxy-4-methoxyl-9,10-dihydrophenylene	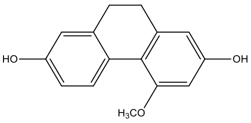	*Pholidota cantonensis*	23.26±1.51% (50 μg/mL)	-	[75]
134.	cis-3,3′-Dihydroxy-5-	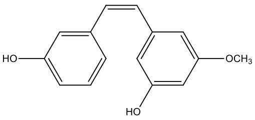	*Pholidota cantonensis*	33.76±0.38% (50 μg/mL)	-	[75]

	methoxystil-bene					
135.	Thunalbene	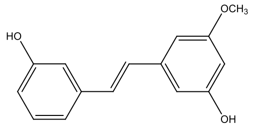	*Pholidota cantonensis*	37.23±0.82% (50 μg/mL)	-	[75]
136.	Batatasin III	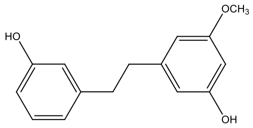	*Pholidota cantonensis*	15.95±0.25% (50 μg/mL)	-	[75]
137.	1,3,5-Trimethoxybenzene	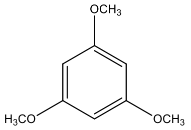	*Pholidota cantonensis*	99.59±0.43% (50 μg/mL)	-	[75]
138.	Phocantol	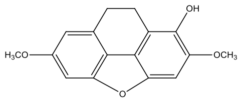	*Pholidota cantonensis*	58.58±1.98% (50 μg/mL)	-	[75]
139.	3,5-Dimethoxy-3′-hydroxybibenzyl	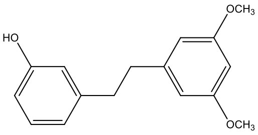	*Pholidota cantonensis*	51.21±0.99% (50 μg/mL)	-	[75]
140.	Deoxyrhapontigenin	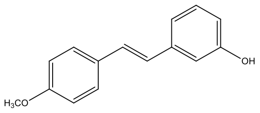	*Rheum lhasaense*	463.07±17.46 μM (IC_{50})	-	[60]
141.	Desoxyrhaponticin	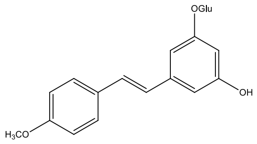	*Rheum lhasaense*	912.97±78.51 μM (IC_{50})	-	[60]
142.	4′-methoxy-scirpusin A	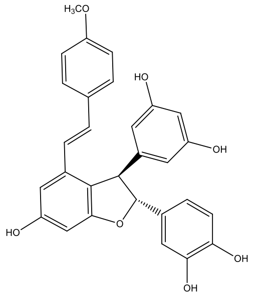	*Rheum lhasaense*	2.18±0.67 μM (IC_{50})	-	[60]
143.	Piceatannol-3′-O-glucoside	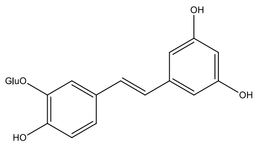	*Rheum lhasaense*	287.83±69.32 μM (IC_{50})	-	[60]

144.	ε-viniferin	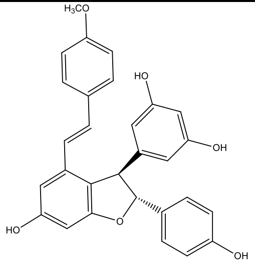	*Rheum lhasaense*	113.73±11.01 μM (IC_{50})	-	[60]
145.	Polydatin	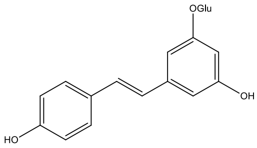	*Rheum lhasaense*	694.13±150.62 μM (IC_{50})	-	[60]
146.	Piceatannol-3′-O-[2″-(3,5-dihydroxy-4-methoxybenzoyl)]-β-d-glucopyranoside	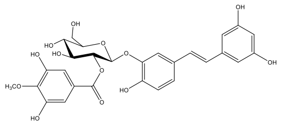	*Rheum lhasaense*	38.93±1.66 μM (IC_{50})	-	[60]
147.	Piceatannol-3′-O-(2″-galloyl)- β-d-glucopyranoside	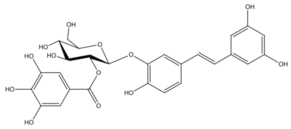	*Rheum lhasaense*	45.18±8.83 μM (IC_{50})	-	[60]
148.	Martynoside	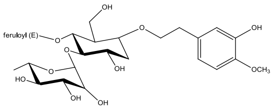	*Sideritis germanicopolitana*	20.75±0.50% (250 μg/mL)	12.08±0.33% (250 μg/mL)	[32]
149.	Leucoseptoside A	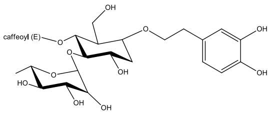	*Sideritis germanicopolitana*	21.30±0.71% (250 μg/mL)	11.50±0.48% (250 μg/mL)	[32]
150.	Lamalboside	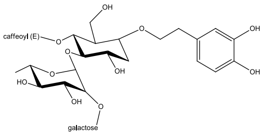	*Sideritis germanicopolitana*	21.37±0.69% (250 μg/mL)	16.92±0.48% (250 μg/mL)	[32]
151.	Decaffeoylverbascoside	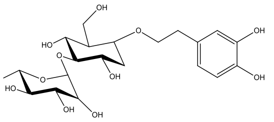	*Sideritis germanicopolitana*	18.35±0.53% (250 μg/mL)	11.05±0.25% (250 μg/mL)	[32]
152.	Xanthomicrol	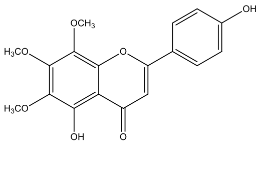	*Sideritis germanicopolitana*	19.47±0.66% (250 μg/mL)	11.36±0.36% (250 μg/mL)	[32]

153.	Isoscutellarein 7-O-[6'''-O-acetyl-β-allopyranosyl-(1→2)]-β-glucopyranoside	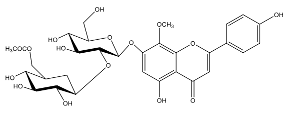	*Sideritis germanicopolitana*	18.47±0.41% (250 μg/mL)	12.91±0.39% (250 μg/mL)	[32]
154.	4'-O-methylisoscutellarein 7-O-[6'''-O-acetyl-β-allopyranosyl-(1→2)]-β-glucopyranoside	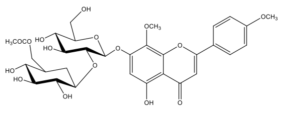	*Sideritis germanicopolitana*	27.90±0.68% (250 μg/mL)	16.32±0.29% (250 μg/mL)	[32]
155.	3'-hydroxy-4'-O-methylisoscutellarein 7-O-[6'''-O-acetyl-β-allopyranosyl-(1→2)]-β-glucopyranoside	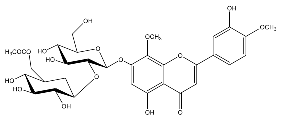	*Sideritis germanicopolitana*	22.79±0.78% (250 μg/mL)	19.41±0.63% (250 μg/mL)	[32]
156.	Dehydrodiconiferylalcohol 4-O-β-D-glucopyranose	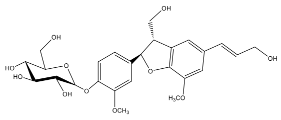	*Sideritis germanicopolitana*	24.32±0.57% (250 μg/mL)	14.28±0.41% (250 μg/mL)	[32]
157.	Pinoresinol 4'-O-β-glucopyranoside	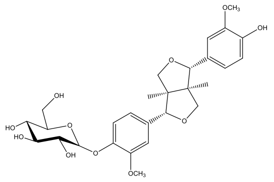	*Sideritis germanicopolitana*	20.17±0.44% (250 μg/mL)	11.02±0.35% (250 μg/mL)	[32]
158.	o-hydroxybenzoic acid	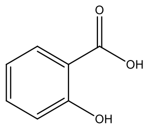	*Bassia indica*	48.5% (250 μg/mL)	-	[34]
159.	p-hydroxybenzoic acid	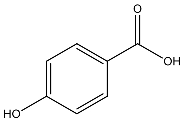	*Bassia indica*	203.2±4.5 μg/mL (IC_{50})	-	[34]

160.	caffeic acid	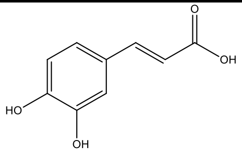	*Bassia indica*	112.9±3.08 μg/mL (IC_{50})	-	[34]
161.	tachioside	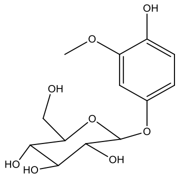	*Bassia indica*	26% (250 μg/mL)	-	[34]
162.	isorhamnetin-3-O-β-D-glucoside	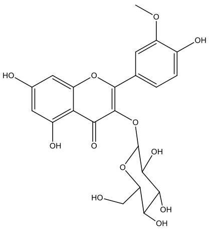	*Bassia indica*	93.2±1.2 μg/mL (IC_{50})	-	[34]
163.	kaempferol-3-O-rutinoside	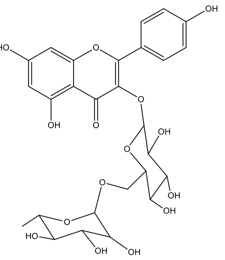	*Bassia indica*	27.9±0.8 μg/mL (IC_{50})	-	[34]
164.	kaempferol-3-O-β-Dglucopyranosyl-(1→6)-O-[β-D-galactopyranosyl-(1→3)-2-O-trans-feruloyl-α-L-rhamnopyranosyl-(1→2)]-β-D-glucopyranoside	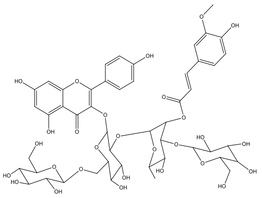	*Bassia indica*	40.6% (250 μg/mL)	-	[34]
165.	isorhamnetin-3-O-β-D-glucopyranosyl-(1→6)-O-[α-Lrhamnopyranosyl-(1→2)]-β-D-glucopyranoside	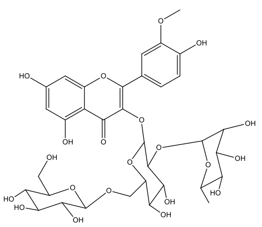	*Bassia indica*	35% (250 μg/mL)	-	[34]

166.	Oleracone J	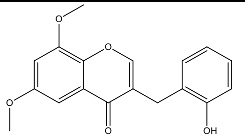	*Portulaca oleracea*	59.08±0.05 μM (IC_{50})	-	[76]
167.	Oleracone K	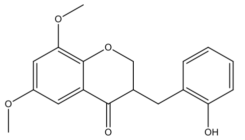	*Portulaca oleracea*	67.89 ± 0.09 μM (IC_{50})	-	[76]
168.	Leucosceptoside A	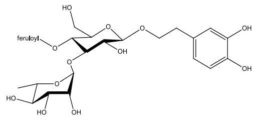	*Citharexylum spinosum*	-	72.85±1.14 μM (IC_{50})	[36]
169.	Martynoside	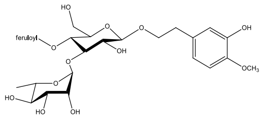	*Citharexylum spinosum*	-	58.77±2.00 μM (IC_{50})	[36]
170.	Isoverbascoside	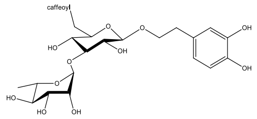	*Citharexylum spinosum*	-	48.47±1.80 μM (IC_{50})	[36]
171.	Plantainoside C	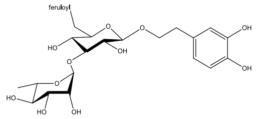	*Citharexylum spinosum*	-	28.44±0.86 μM (IC_{50})	[36]
172.	4-hydroxy-2,6-dimethoxyphenyl 6′-O-vanilloyl-β-D-glucopyranoside	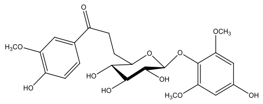	*Citharexylum spinosum*	-	117.65±2.46 μM (IC_{50})	[36]
173.	Plucheoside D_1	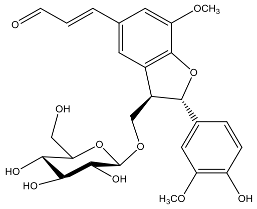	*Citharexylum spinosum*	-	98.14±2.72 μM (IC_{50})	[36]
174.	Plucheoside D_2	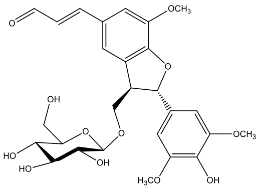	*Citharexylum spinosum*	-	71.28 ± 2.82 μM (IC_{50})	[36]
175.	Tyrosol	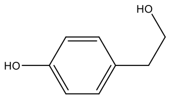	*Citharexylum spinosum*	-	120.18±1.90 μM (IC_{50})	[36]

176.	quercetin-3-O-[2''-O-(6'''-O-E-feruloyl)-β-D-glucopyranosyl]-β-D-glucopyranoside	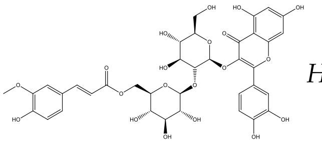	*Hedyotis diffusa*	46.22 ± 1.59 μM (IC_{50})	13.77 ± 0.37 μM (IC_{50})	[37]
177.	6''-O-p-hydroxybenzoyl-davidioside	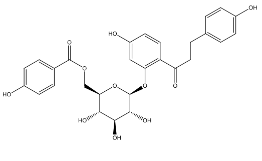	*Viburnum davidii*	36.883±3.021 μM (IC_{50})	39.274±0.491 μM (IC_{50})	[77]
178.	Davidioside	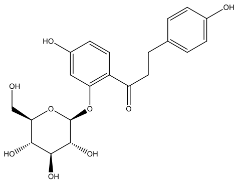	*Viburnum davidii*	39.504±1.121 μM (IC_{50})	43.101±0.512 μM (IC_{50})	[77]
179.	ciquitin A	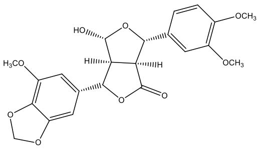	*Leucophyllum ambiguum*	3 nM (IC_{50})	-	[78]
180.	ciquitin B	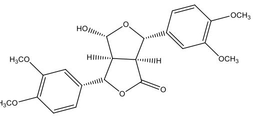	*Leucophyllum ambiguum*	28 nM (IC_{50})	-	[78]
181.	ciquitin D	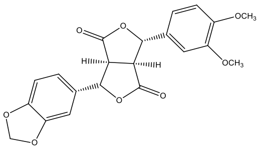	*Leucophyllum ambiguum*	158 nM (IC_{50})	-	[78]
182.	3-methoxy-kobusin	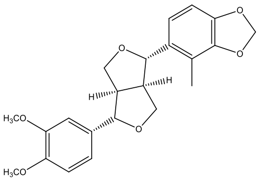	*Leucophyllum ambiguum*	93 nM (IC_{50})	-	[78]
183.	sasertemin	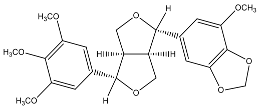	*Leucophyllum ambiguum*	0.46 μM (IC_{50})	-	[78]
184.	yangambin	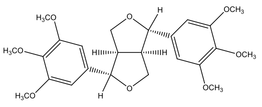	*Leucophyllum ambiguum*	0.616 μM (IC_{50})	-	[78]
185.	magnolin	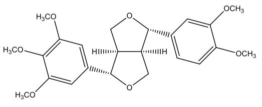	*Leucophyllum ambiguum*	0.903 μM (IC_{50})	-	[78]

186.	ciquitin C	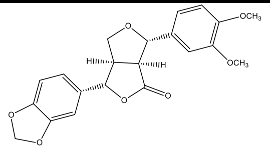	*Leucophyllum ambiguum*	2.229 µM (IC_{50})	-	[78]
187.	Garcinia biflavonoid 2	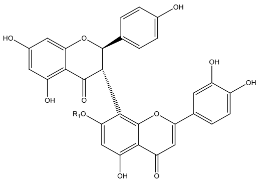	*Garcinia fusca*	-	16.75±0.23 µM (IC_{50})	[67]
188.	(+) gallocatechin	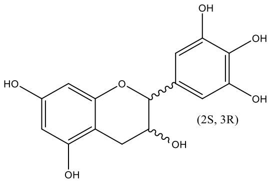	*Kadsura coccinea*	46.78±1.92 µM (IC_{50})	-	[40]
189.	(−)-epicatechin	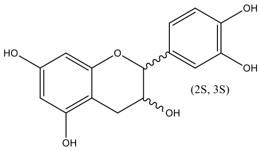	*Kadsura coccinea*	68.28±1.11 µM (IC_{50})	-	[40]
190.	Icariside E_3	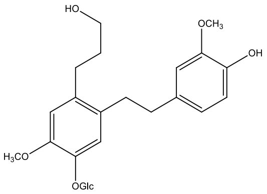	*Kadsura coccinea*	59.59±1.62 µM (IC_{50})	-	[40]
191.	(−)-secoisolariciresinol-9-Oβ-D-xylopyranoside	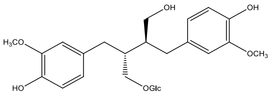	*Kadsura coccinea*	59.14±3.84 µM (IC_{50})	-	[40]
192.	cimidahurinine	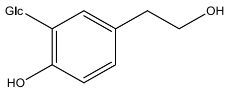	*Kadsura coccinea*	87.20±1.12 µM (IC_{50})	-	[40]
193.	salidroside	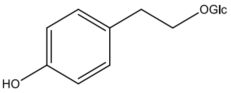	*Kadsura coccinea*	98.75±2.20 µM (IC_{50})	-	[40]
194.	chlorogenic acid	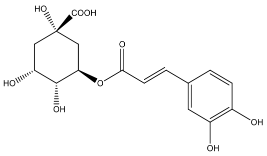	*Kadsura coccinea*	57.58±2.45 µM (IC_{50})	-	[40]
195.	3-methoxyquercetin	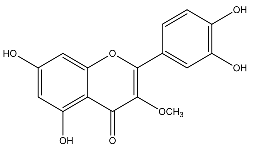	*Cassia timorensis*	83.71±4.67 µM (IC_{50})	-	[79]

196.	quercetagetin7-O-(6-O-caffeoyl-β-D-glucopyranoside)	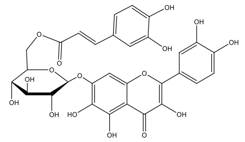	*Eupatorium adenophorum*	12.08±0.42 μg/mL (IC_{50})	-	[50]
197.	quercetagetin-7-O-(6-O-p-coumaroyl-β-glucopyranoside)	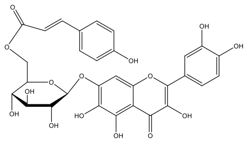	*Eupatorium adenophorum*	15.01±0.01 μg/mL (IC_{50})	-	[50]
198.	4′-methyl quercetagetin 7-O-(6″-O-E-caffeoyl glucopyranoside)	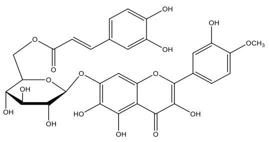	*Eupatorium adenophorum*	17.12±0.40 μg/mL (IC_{50})	-	[50]
199.	5,4′-Dihydroxytlavone	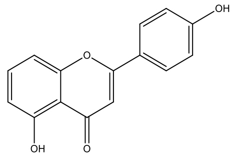	*Eupatorium adenophorum*	28.63±1.66 μg/mL (IC_{50})	-	[50]
200.	chrysoeriol	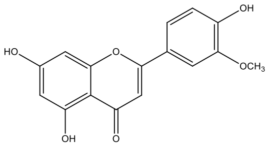	*Eupatorium adenophorum*	49.27±0.40 μg/mL (IC_{50})	-	[50]
201.	chrysoplenetin	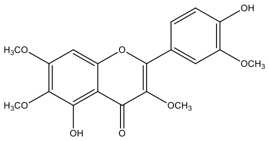	*Eupatorium adenophorum*	86.01±2.52 μg/mL (IC_{50})	-	[50]
202.	quercetin-3-O-β-D-glucopyranoside	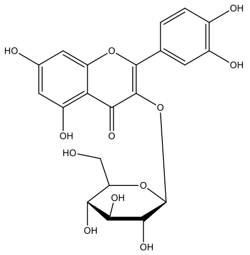	*Eupatorium adenophorum*	54.94±0.16 μg/mL (IC_{50})	-	[50]
203.	fortunellin	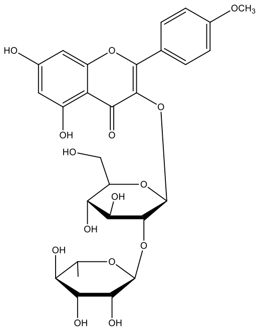	*Eupatorium adenophorum*	67.56±0.13 μg/mL (IC_{50})	-	[50]

204.	3-hydroxy-phloridzin	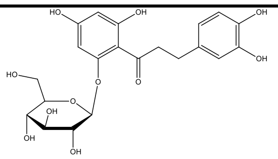	*Eupatorium adenophorum*	49.67±0.06 μg/mL (IC_{50})	-	[50]
205.	methylenebisphloridzin	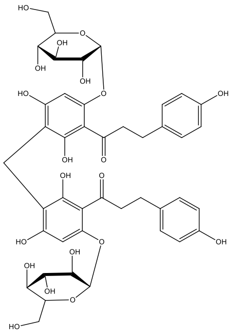	*Eupatorium adenophorum*	50.19±0.79 μg/mL (IC_{50})	-	[50]
206.	helichrysin A	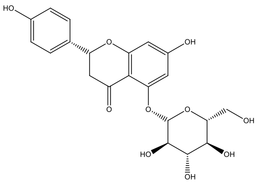	*Helichrysum plicatum*	1.69 μM (IC_{50})	2.27 μM (IC_{50})	[48]
207.	helichrysin B	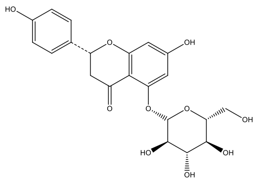	*Helichrysum plicatum*	2.90 μM (IC_{50})	3.89 μM (IC_{50})	[48]
208.	isosalipurposide	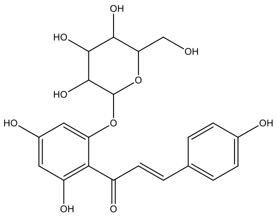	*Helichrysum plicatum*	2.53 μM (IC_{50})	3.78 μM (IC_{50})	[48]
209.	Liquiritin apioside	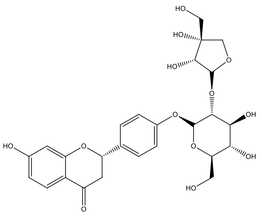	*Glycyrrhiza uralensis*	36.68±1.42 μM (IC_{50})	>40 μM (IC_{50})	[80]
210.	Knepachycarpic acid A	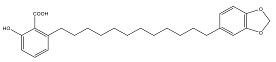	*Knema pachycarpa*	8.19±0.63 μM (IC_{50})	-	[81]
211.	Knepachycarpic acid B	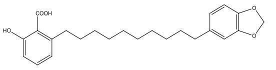	*Knema pachycarpa*	3.89±0.33 μM (IC_{50})	-	[81]
212.	Knepachycarpanol A	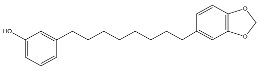	*Knema pachycarpa*	2.60±0.24 μM (IC_{50})	-	[81]
213.	Knepachycarpanol B	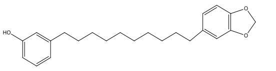	*Knema pachycarpa*	7.09±0.59 μM (IC_{50})	-	[81]

214.	Knepachy-carpasinol	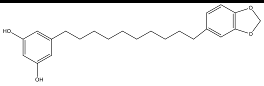	*Knema pachycarpa*	2.46±0.23 μM (IC_{50})	-	[81]
215.	licarin A	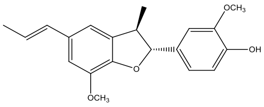	*Myristica fragrans*	111.3±0.07 μg/mL (IC_{50})	-	[62]
216.	Glabrateph-rinol	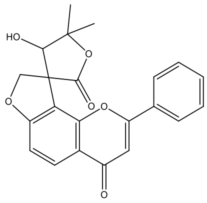	*Tephrosia purpurea* subsp. *dunensis*	4.31±0.75 μM (IC_{50})	-	[82]
217.	garbogiol	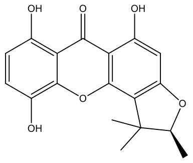	*Garcinia fusca*	23.90±0.59 μM (IC_{50})	14.04±0.66 μM (IC_{50})	[67]
218.	3-methox-ycowaxan-thone	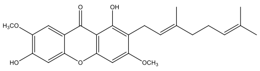	*Garcinia fusca*	73.15±0.32 μM (IC_{50})	108.28±0.47 μM (IC_{50})	[67]
219.	rheediaxan-thone A	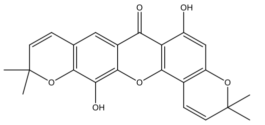	*Garcinia fusca*	-	126.42±0.19 μM (IC_{50})	[67]
220.	cowanin	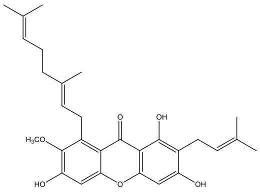	*Garcinia fusca*	1.09±0.09 μM (IC_{50})	0.51±0.006 μM (IC_{50})	[67]
221.	cowaxan-thone	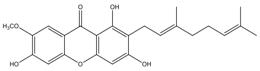	*Garcinia fusca*	3.89±0.15 μM (IC_{50})	4.25±1.09 μM (IC_{50})	[67]
222.	Cowagarci-none E	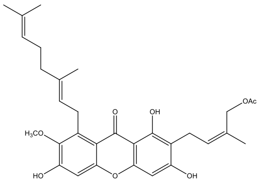	*Garcinia fusca*	0.79±0.05 μM (IC_{50})	0.048±0.003 μM (IC_{50})	[67]
223.	Norcowanin	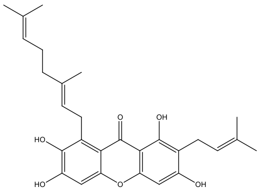	*Garcinia fusca*	0.33±0.04 μM (IC_{50})	0.35±0.03 μM (IC_{50})	[67]
224.	Cowanol	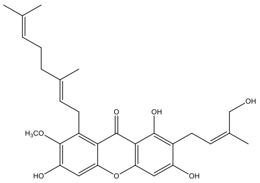	*Garcinia fusca*	0.72±0.05 μM (IC_{50})	1.84±0.29 μM (IC_{50})	[67]

225.	Fuscaxanthone M	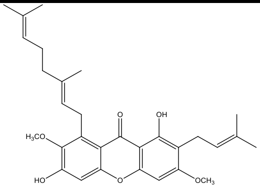	*Garcinia fusca*	97.22±0.26 μM (IC_{50})	42.95±0.53 μM (IC_{50})	[67]
226.	Gartanin	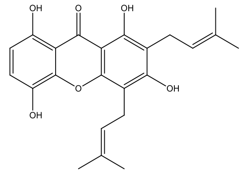	*Garcinia fusca*	9.35±0.0003 μM (IC_{50})	1.46±0.00003 μM (IC_{50})	[67]
227.	8-desoxygartanin	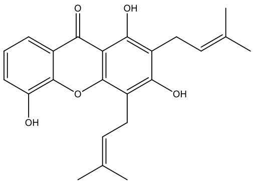	*Garcinia fusca*	20.41±0.14 μM (IC_{50})	1.23±0.00003 μM (IC_{50})	[67]
228.	β-mangostin	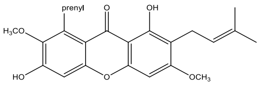	*Garcinia fusca*	-	82.00±0.60 μM (IC_{50})	[67]
229.	7-methoxygarcinone E	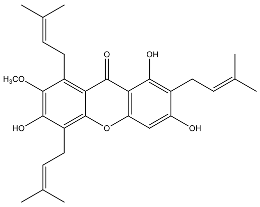	*Garcinia fusca*	10.95±0.13 μM (IC_{50})	2.92±0.06 μM (IC_{50})	[67]
230.	fuscaxanthone A	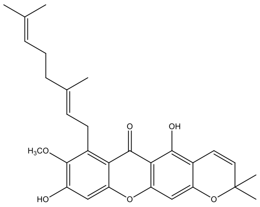	*Garcinia fusca*	81.26±5.9 μM (IC_{50})	25.67±0.23 μM (IC_{50})	[67]
231.	Helminthosporin	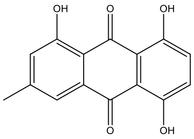	*Rumex abyssinicus*	2.63±0.09 μM (IC_{50})	2.99±0.55 μM (IC_{50})	[83]
232.	Emodin	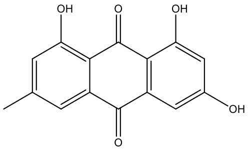	*Rumex abyssinicus*	15.21±3.52 μM (IC_{50})	-	[83]
233.	Chrysophanol	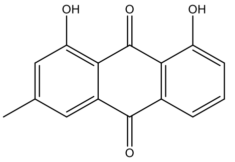	*Rumex abyssinicus*	33.7±1.83 μM (IC_{50})	-	[83]
234.	Physcion	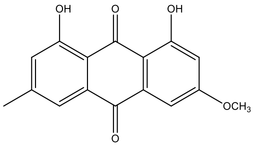	*Rumex abyssinicus*	12.16±0.36 μM (IC_{50})	-	[83]

235.	4,8-Dihydroxytetralone	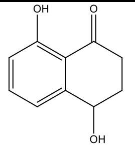	*Carya illinoinensis*	101.48±4.00 μg/mL (IC_{50})	-	[84]
236.	(+)-Ligballinol	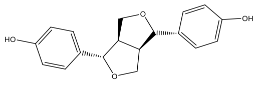	*Momordica Charantia*	-	32.2±0.2 % (50 μM)	[85]

Table S3: Coumarins as Acetyl and Butyryl cholinesterase inhibitors

Number	Name	Structure	Plant source	IC_{50} or %inh (conc.)		Reference
				AChE	BChE	
1.	Pteryxin	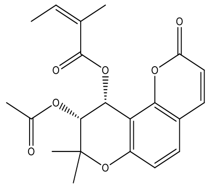	*Mutellina purpurea*	-	12.96 ± 0.70 mg/ml (IC_{50})	[86]
2.	psoralen	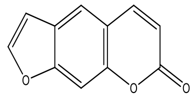	*Caryopteris odorata*	-	312.53±0.28 μM (IC_{50})	[87]
3.	methoxsalen	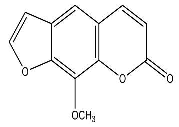	*Caryopteris odorata*	-	203.72±0.27 μM (IC_{50})	[87]
4.	isoimperatorin	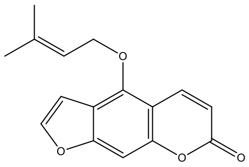	*Caryopteris odorata*	-	263.52±0.12 μM (IC_{50})	[87]
			Angelica archangelica	>250 μM (IC_{50})	>250 μM (IC_{50})	[88]
5.	oxypeucedanin	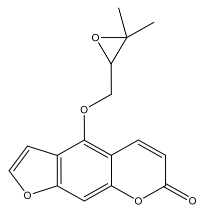	*Caryopteris odorata*	-	250.11±0.36 μM (IC_{50})	[87]
			Angelica purpurascens	19.3±1.87% (20 μg/mL)	36.89±1.23% (20 μg/mL)	[89]
6.	bergamottin	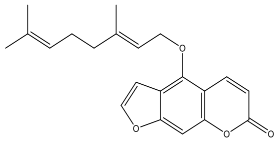	*Caryopteris odorata*	-	234.42±0.58 μM (IC_{50})	[87]
7.	7-Isopentenyloxycoumarin	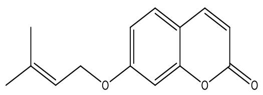	*Semi synthetic*	12.51 ± 2.83 % (100 μM)	11.77 ± 0.89 μM (IC_{50})	[90]

8.	Auraptene	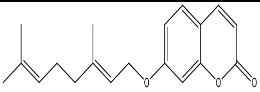	*Semi synthetic*	6.75 ± 2.20 % (100 µM)	12.68 ± 2.23 µM (IC_{50})	[90]
9.	Umbelliprenin	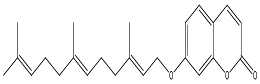	*Semi synthetic*	-	26.66 ± 1.11 % (100 µM)	[90]
			Heptaptera cilicica	5.86 ± 0.030 µM (IC_{50})	1.10 ± 0.190 µM (IC_{50})	[91]
10.	7-Isopentenyloxy-4-methylcoumarin	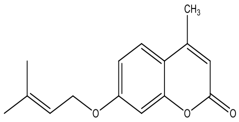	*Semi synthetic*	14.92 ± 0.60 % (100 µM)	8.18 ± 0.74 µM (IC_{50})	[90]
11.	7-Isopentenyloxycoumarin-3-carboxylic acid	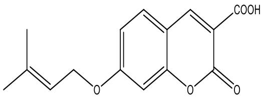	*Semi synthetic*	-	10.45 ± 2.92 % (100 µM)	[90]
12.	3-Acetyl-7-isopentenyloxycoumarin	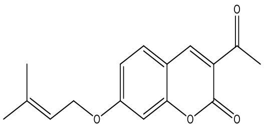	*Semi synthetic*	31.14 ± 2.59 % (100 µM)	11.75 ± 1.82 µM (IC_{50})	[90]
13.	3-Acetyl-7-geranyloxycoumarin	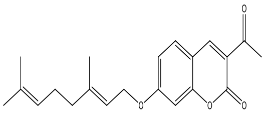	*Semi synthetic*	–	16.29 ± 2.03 % (100 µM)	[90]
14.	3-Acetyl-7-farnesyloxycoumarin	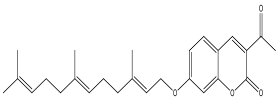	*Semi synthetic*	6.14 ± 2.11 % (100 µM)	17.36 ± 2.71 % (100 µM)	[90]
15.	3-Chloro-7-isopentenyloxy-4-methylcoumarin	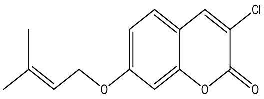	*Semi synthetic*	11.47 ± 1.73 % (100 µM)	15.56 ± 2.85 % (100 µM)	[90]
16.	3-Chloro-7-geranyloxy-4-methylcoumarin	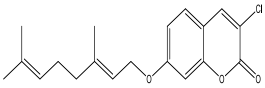	*Semi synthetic*	7.03 ± 2.08 % (100 µM)	16.64 ± 2.64 % (100 µM)	[90]
17.	7-Geranyloxy-4-methylcoumarin	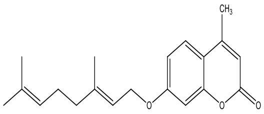	*Semi synthetic*	-	51.04 ± 1.88 % (100 µM)	[90]
18.	3-Chloro-7-farnesyloxy-4-methylcoumarin	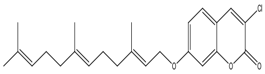	*Semi synthetic*	17.23 ± 2.08 % (100 µM)	23.77 ± 2.19 % (100 µM)	[90]
19.	7-Farnesyloxy-4-methylcoumarin	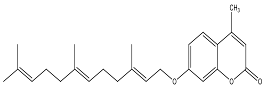	*Semi synthetic*	-	23.82 ± 2.41 % (100 µM)	[90]
20.	umbelliprenin-10′,11′-monoepoxide	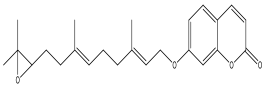	*Heptaptera cilicica*	> 100 µM	12.59 ± 0.021 µM (IC_{50})	[91]
21.	6,7-Diisopentenyloxycoumarin	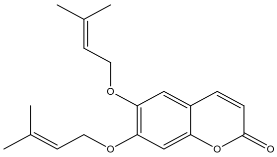	*Semi synthetic*	40.39 ± 3.81 % (100 µM)	–	[90]

22.	6,7-Di-geranyloxycoumarin	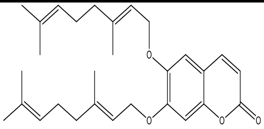	*Semi synthetic*	11.70 ± 3.99 % (100 µM)	7.01 ± 2.73 % (100 µM)	[90]
23.	6,7-Difarnesyloxycoumarin	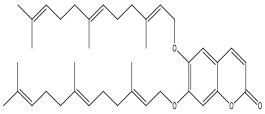	*Semi synthetic*	21.33 ± 0.55 % (100 µM)	21.35 ± 1.53 % (100 µM)	[90]
24.	7-Ethoxycoumarin	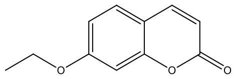	*Semi synthetic*	9.76 ± 1.92 % (100 µM)	83.65 ± 2.39 µM (IC_{50})	[90]
25.	7-(2′-Butenyloxy)coumarin	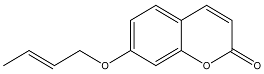	*Semi synthetic*	9.75 ± 2.09 % (100 µM)	16.49 ± 0.99 µM (IC_{50})	[90]
26.	7-Styryloxycoumarin	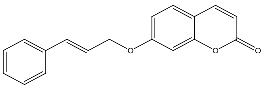	*Semi synthetic*	26.72 ± 1.93 % (100 µM)	7.01 ± 0.28 µM (IC_{50})	[90]
27.	7-(2′,2′-Dimethyl)-n propoxycoumarin	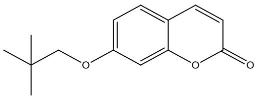	*Semi synthetic*	-	26.77 ± 1.35 % (100 µM)	[90]
28.	7-Methoxycoumarin	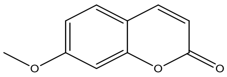	*Semi synthetic*	4.32 ± 1.01 % (100 µM)	46.53 ± 2.39 % (100 µM)	[90]
29.	7-(2′-Pentinyloxy) coumarin	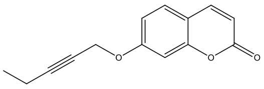	*Semi synthetic*	12.01 ± 1.64 % (100 µM)	18.48 ± 0.73 µM (IC_{50})	[90]
30.	7-(3′-Methyl)-n butyloxy) coumarin	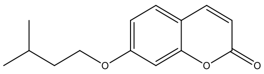	*Semi synthetic*	8.22 ± 0.88 % (100 µM)	23.32 ± 0.57 µM (IC_{50})	[90]
31.	7-Allyloxycoumarin	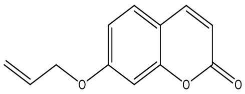	*Semi synthetic*	11.21 ± 2.37 % (100 µM)	43.31 ± 3.63 µM (IC_{50})	[90]
32.	7-n Propoxycoumarin	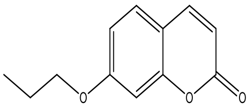	*Semi synthetic*	11.40 ± 1.20 % (100 µM)	39.89 ± 3.90 µM (IC_{50})	[90]
33.	Imperatorin	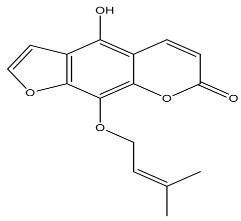	*Angelica Officinalis*	46.11 ± 0.92 % (100µg/mL)	83.9±80.99 % (100µg/mL)	[92]
34.	phellopterin	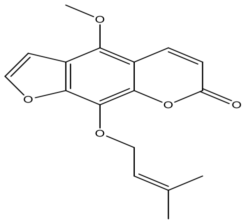	*Angelica archangelica*	>250 µM (IC_{50})	>250 µM (IC_{50})	[88]

35.	Xanthotoxin	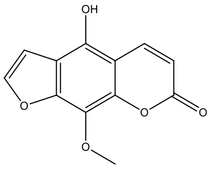	*Angelica archangelica*	156 ± 15 µM (IC$_{50}$)	14.4 ± 3.2 µM (IC$_{50}$)	[88]
36.	Bergapten	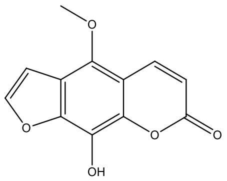	*Angelica officinalis*	66.08±2.88 % (100 µg/mL)	88.0±40.83 % (100 µg/mL)	[92]
			Angelica purpurascens	18.98±2.98% (20 µg/mL)	31.00±3.0% (20 µg/mL)	[89]
37.	isopimpinelin	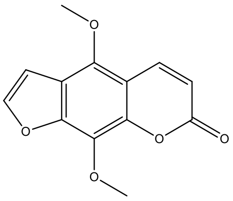	*Angelica archangelica*	>250 µM (IC$_{50}$)	>250 µM (IC$_{50}$)	[88]
			Angelica officinalis	32.65±6.10 % (25 µg/mL)	86.69±2.56 % (25 µg/mL)	[92]
38.	Pimpinellin	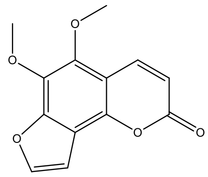	*Angelica archangelica*	>250 µM (IC$_{50}$)	>250 µM (IC$_{50}$)	[88]
			Leiotulus dasyanthus	18. 98 ± 2.98 % (20 µg/mL)	31.00 ± 3.02 % (20 µg/mL)	[45]
39.	heraclenol-2’-O-angelate	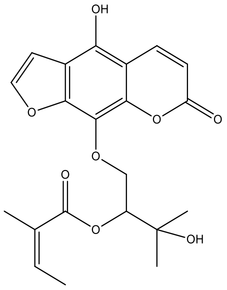	*Angelica archangelica*	>1000 µM (IC50)	7.5±1.8 µM (IC$_{50}$)	[88]

40.	byakangelicin-2’-O-angelate	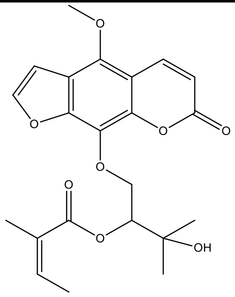	*Angelica archangelica*	>1000 µM (IC_{50})	>1000 µM (IC_{50})	[88]
41.	byakangelicin-2’-O-isovalerate	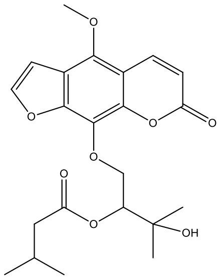	*Angelica archangelica*	>1000 µM (IC_{50})	>1000 µM (IC_{50})	[88]
42.	Umbelliferone	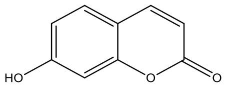	*Angelica archangelica*	>250 µM (IC_{50})	>250 µM (IC_{50})	[88]
			Leiotulus dasyanthus	23.54 ± 1.29 % (20 µg/mL)	66.55 ± 2.61 % (20 µg/mL)	[45]
			Leiotulus dasyanthus	61.09 ± 4.46 % (20 µg/mL)	40.99 ± 5.61 % (20 µg/mL)	[45]
43.	osthol	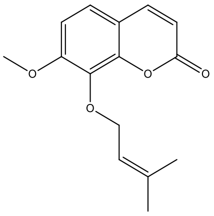	*Angelica archangelica*	>250 µM (IC_{50})	>250 µM (IC_{50})	[88]
44.	conferone	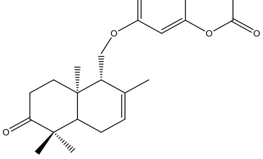	*Heptaptera cilicica*	3.31 ± 0.014 µM (IC_{50})	9.31 ± 0.280 µM (IC_{50})	[91]
45.	mogoltacin	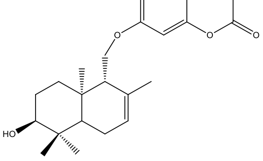	*Heptaptera cilicica*	1.95 ± 0.050 µM (IC_{50})	9.74 ± 0.003 µM (IC_{50})	[91]
46.	feselol	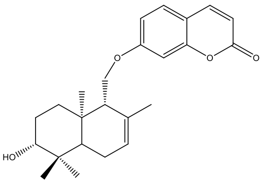	*Heptaptera cilicica*	1.26 ± 0.010 µM (IC_{50})	9.98 ± 0.240 µM (IC_{50})	[91]

47.	Daphnetin	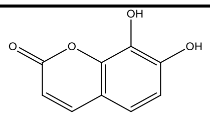	*Portulaca oleracea*	72.16 ± 0.28 µM (IC_{50})	-	[29]
48.	Esculetin	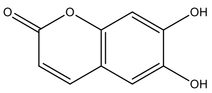	*Portulaca oleracea*	71.50 ± 0.39 µM (IC_{50})	-	[29]
49.	Scopoletin	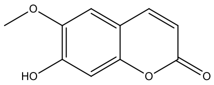	*Croton oligandrus*	-	79.2±0.26 µM (IC_{50})	[72]
			Argyreia speciosa	5.34 µM (IC_{50})	9.11 µM (IC_{50})	[93]
50.	6,7-dihydroxycoumarin	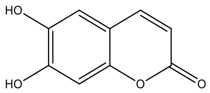	*Bassia indica*	3.6±0.07 µg/mL (IC_{50})	-	[34]
51.	Kamonol acetate	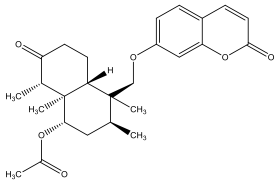	*Ferula pseudalliacea*	63.9 µM (IC_{50})	-	[94]
52.	3′,4′-disenecioylkhellactone	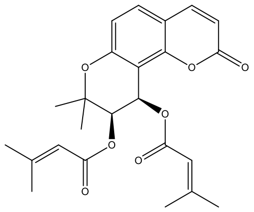	*Peucedanum japonicum*	21.3±7.69 µM (IC_{50})	10.7±0.060 µM (IC_{50})	[95]
53.	senecioyl4′-angeloyl-khellactone	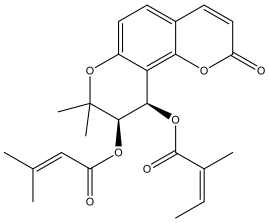	*Peucedanum japonicum*	>40 µM (IC_{50})	7.20±0.79 µM (IC_{50})	[95]
54.	calipteryxin	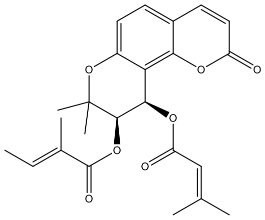	*Peucedanum japonicum*	25.6±4.50 µM (IC_{50})	>40 µM (IC_{50})	[95]
55.	Anomalin	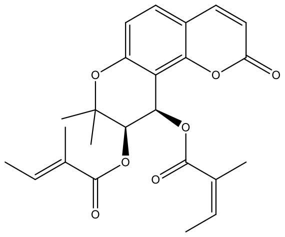	*Peucedanum japonicum*	17.9±5.59 µM (IC_{50})	>40 µM (IC_{50})	[95]

56.	3′-senecioyl-4′-isovalerylkhellactone	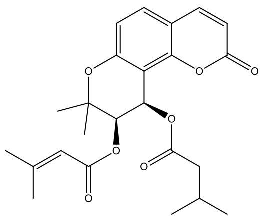	*Peucedanum japonicum*	31.6±4.40 µM (IC_{50})	>40 µM (IC_{50})	[95]
57.	3′-isovaleryl-4′-senecioylkhellactone	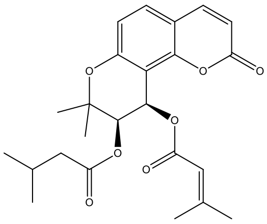	*Peucedanum japonicum*	36.1±0.66 µM (IC_{50})	12.5±2.82 µM (IC_{50})	[95]
58.	3′-senecioyl-4′-(2-methylbutyryl)khellactone	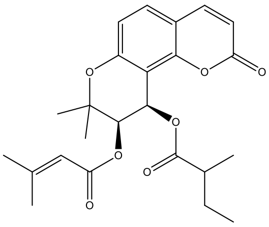	*Peucedanum japonicum*	>40 µM (IC_{50})	10.2±2.25 µM (IC_{50})	[95]
59.	3′-isovaleryl-4′-angeloylkhellactone	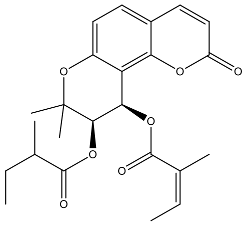	*Peucedanum japonicum*	29.0±1.15 µM (IC_{50})	>40 µM (IC_{50})	[95]
60.	3′-angeloyl-4′-(2-methylbutyryl) khellactone	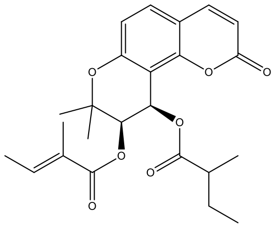	*Peucedanum japonicum*	9.28±0.094 µM (IC_{50})	>40 µM (IC_{50})	[95]
61.	3′,4′-diisovalerylkhellactone	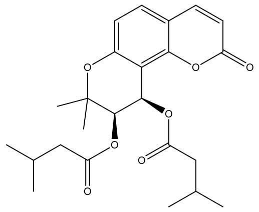	*Peucedanum japonicum*	28.1±0.33 µM (IC_{50})	>40 µM (IC_{50})	[95]

62.	3′-isovaleryl-4′-(2-methyl-butyroyl)khellactone	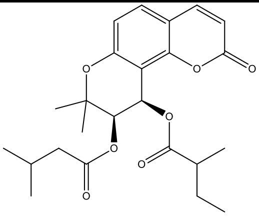	*Peucedanum japonicum*	10.0±0.48 µM (IC_{50})	>40 µM (IC_{50})	[95]

Table S4: Other Acetyl and Butyryl cholinesterase inhibitors

Number	Name	Structure	*Plant source*	IC_{50} or %inh. (conc.)		Reference
				AChE	BChE	
1.	dioctyl phthalate	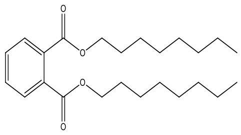	*Lonicera quinquelocularis*	8.74 ± 0.07 mg/mL (IC_{50})	20.12 ± 0.079 mg/mL (IC_{50})	[96]
2.	Bis (7-acetoxy-2-ethyl-5-methylheptyl) phthalate	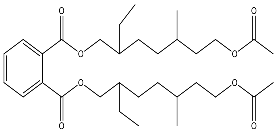	*Lonicera quinquelocularis*	1.65 ± 0.03 mg/mL (IC_{50})	5.98 ± 0.079 mg/mL (IC_{50})	[96]
3.	Neopentyl-4-ethoxy-3, 5-bis [3-methyl-2-butenyl] benzoate	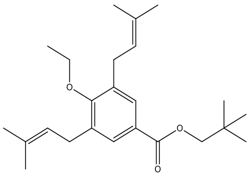	*Lonicera quinquelocularis*	5.27 ± 0.04 mg/mL (IC_{50})	14.76 ± 0.087 mg/mL (IC_{50})	[96]
4.	Neopentyl-4-hydroxy-3, 5-bis [3-methyl-2-butenyl] benzoate	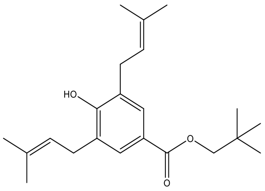	*Lonicera quinquelocularis*	3.43 ± 0.02 mg/mL (IC_{50})	9.84 ± 0.037 mg/mL (IC_{50})	[96]
5.	eluptol	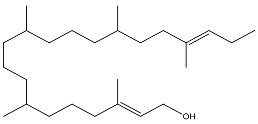	*Pycnanthus angolensis*	22.26 µg/ml (IC_{50})	34.61 µg/ml (IC_{50})	[97]
6.	omifoate A	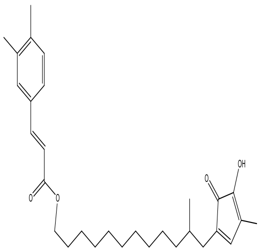	*Pycnanthus angolensis*	6.51 µg/ml (IC_{50})	9.07 µg/ml (IC_{50})	[97]

7.	Chuanxiongdiolide A	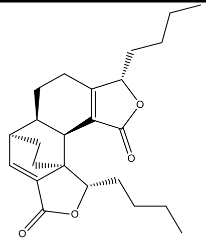	*Ligusticum chuanxiong*	-	63.0% (50 µM)	[98]
8.	Chuanxiongdiolide B	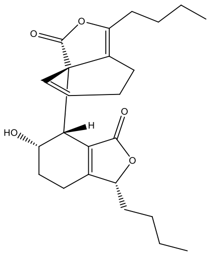	*Ligusticum chuanxiong*	-	21.7% (50 µM)	[98]
9.	(–)-Pteroside N	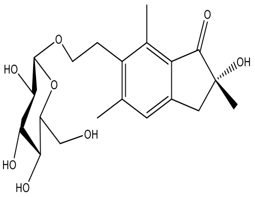	*Pteridium aquilinum*	7.39 ± 0.99 µM (IC_{50})	4.47 ± 0.29 µM (IC_{50})	[99]
10.	pterosinone	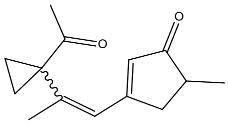	*Pteridium aquilinum*	72.9 ± 0.73 µM (IC_{50})	87.7 ± 1.6 µM (IC_{50})	[99]
11.	β-Sitosterol-3-O-β-D-glucoside	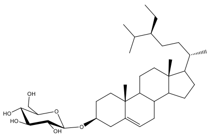	*Thunbergia erecta*	75.72 ± 4.33 ng/mL (IC_{50})	-	[47]
12.	Hysteroside	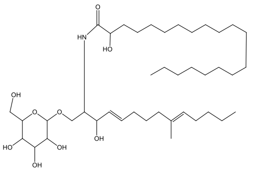	*Hysterangium inflatum*	35.63±0.46% (100 µg/mL)	45.37±0.81% (100 µg/mL)	[100]
13.	Brassicasterol	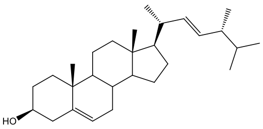	*Hysterangium inflatum*	21.69±0.55% (100 µg/mL)	30.93±0.95% (100 µg/mL)	[100]
14.	Ergosterol	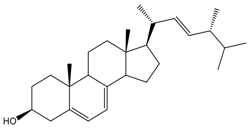	*Hysterangium inflatum*	20.66±0.98% (100 µg/mL)	24.74±1.05% (100 µg/mL)	[100]
15.	Ergosterol D	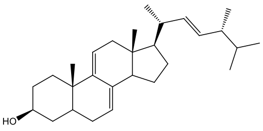	*Hysterangium inflatum*	28.76 ± 0.54% (100 µg/mL)	39.21 ± 0.86% (100 µg/mL)	[100]
16.	Ergosterol peroxide	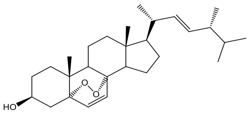	*Hysterangium inflatum*	13.61 ± 0.42% (100 µg/mL)	25.57 ± 1.20% (100 µg/mL)	[100]

17.	Docosanoic acid	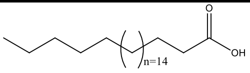	*Grewia optiva*	130 µg/mL (IC_{50})	130 µg/mL (IC_{50})	[30]
18.	Methanetriol mono formate	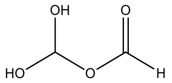	*Grewia optiva*	75 µg/mL (IC_{50})	75 µg/mL (IC_{50})	[30]
19.	2,2'-(1,4-phenylene)bis(3-methylbutanoic acid	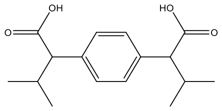	*Grewia optiva*	55 µg/mL (IC_{50})	60 µg/mL (IC_{50})	[30]
20.	Agelorin A	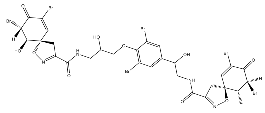	*Suberea clavata*	0.19±0.2	-	[101]
21.	11,17-dideoxyfistularin	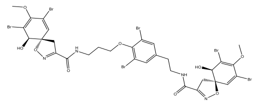	*Suberea clavata*	10±0.3	-	[101]
22.	11-hydroxyaerothionin	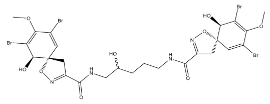	*Suberea clavata*	10±0.3	-	[101]
23.	Geddic acid	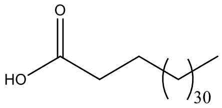	*Croton oligandrus*	-	69.2±0.24 µM (IC_{50})	[72]
24.	β-sitosterol	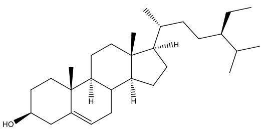	*Croton oligandrus*	-	36.3±0.92 µM (IC_{50})	[72]
			Bassia indica	55.8±3.8 µg/mL (IC_{50})	-	[34]
			Kadsura coccinea	67.37±1.28 µM (IC_{50})	-	[40]
			Helichrysum plicatum	2.59 µM (IC_{50})	2.18 µM (IC_{50})	[48]
25.	Stigmastane-3,6-dione	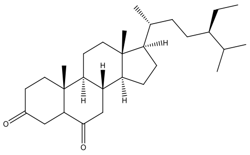	*Croton oligandrus*	-	85.4±0.76 µM (IC_{50})	[72]
26.	3-(R)-acetyl-1-(3′,4′-dihydroxyphenyl)-7-(4″-hydroxy-3″-methoxyphenyl)-heptane	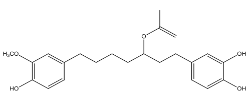	*Carya illinoinensis*	247.64±59.36 µg/mL (IC_{50})	-	[84]
27.	11-Oxo-1,17-epoxy-7-(2-hydroxylphenyl)-13-(16-methoxyphenyl)-heptane	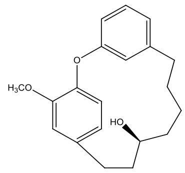	*Carya illinoinensis*	330.60±25.89 µg/mL (IC_{50})	-	[84]
28.	Eloundemnoside	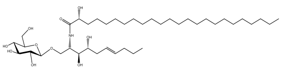	*Celtis adolphi-friderici*	-	66.6±0.92 µM (IC_{50})	[33]

29.	Heptacosanoic acid	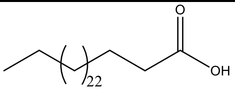	*Celtis adolphi-friderici*	-	45.2±0.73 µM (IC_{50})	[33]
30.	Glycerol-1-octade-canoate	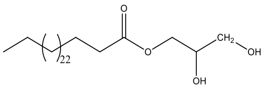	*Celtis adolphi-friderici*	-	61.1±0.51 µM (IC_{50})	[33]
31.	thymidine	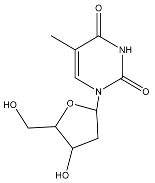	*Bassia indica*	45.7±0.57 µg/mL (IC_{50})	-	[34]
32.	Stigmastadienone	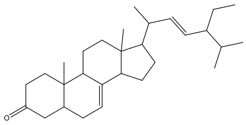	*Isodon rugosus*	13.52 µg/mL (IC_{50})	11.53 µg/mL (IC_{50})	[102]
33.	Cremaphenanthrene F	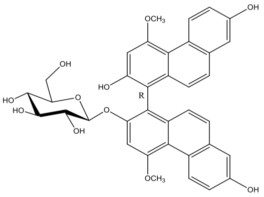	*Cremastra appen-diculata*	>200 µM (IC_{50})	14.62±2.15 µM (IC_{50})	[103]
34.	Cremaphenanthrene G	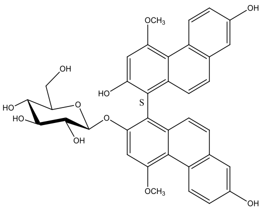	*Cremastra appen-diculata*	>200 µM (IC_{50})	79.56±0.78 µM (IC_{50})	[103]
35.	2,3,4-trimethoxy-7,8-methylenedioxyphe-nanthrene	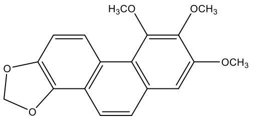	*Dioscorea com-munis*	11.30±0.34% (200 µg/mL)	37.51±2.97% (200 µg/mL)	[104]
36.	2,4-dimethoxy-7,8-methylendioxy-3-phenanthrenol	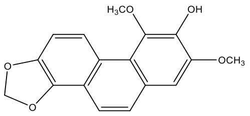	*Dioscorea com-munis*	42.53±0.72% (200 µg/mL)	11.40±0.24 µg/mL (IC_{50})	[104]
37.	2,4,8-trimethoxy-3,7-phenanthrenediol	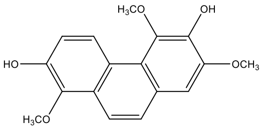	*Dioscorea com-munis*	69.41±2.46 µg/mL (IC_{50})	14.60±0.56 µg/mL (IC_{50})	[104]
38.	Oleraciamide E	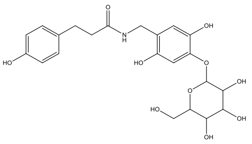	*Portulaca oleracea*	52.43±0.33 µM (IC_{50})	-	[105]
39.	Galactomannan II	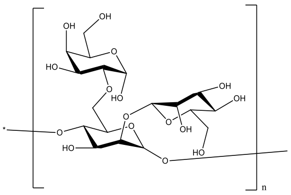	*Ganoderma ad-spersum*	36.71±0.94 µg/mL (IC_{50})	40.18±0.26 µg/mL (IC_{50})	[106]

40.	Macrolobin	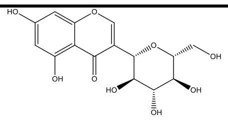	*Macrolobium latifolium*	0.80 μM (IC_{50})	-	[107]
41.	Spinasterol	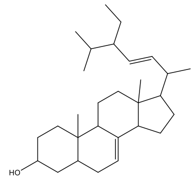	*Acacia auriculiformis*	44.19±2.59 μg/mL (IC_{50})	-	[108]
42.	Seladelicatulasine A	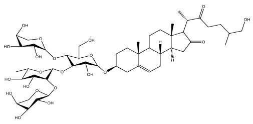	*Selaginella delicatula*	0.31±0.060 μM (IC_{50})	0.37±0.145 μM (IC_{50})	[109]
43.	Seladelicatulasine B	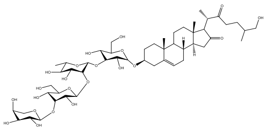	*Selaginella delicatula*	0.09±0.014 μM (IC_{50})	2.01±0.005 μM (IC_{50})	[109]
44.	Seladelicatulasine C	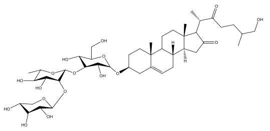	*Selaginella delicatula*	5.86±1.213 μM (IC_{50})	20.65±3.376 μM (IC_{50})	[109]
45.	Seladelicatulasine D	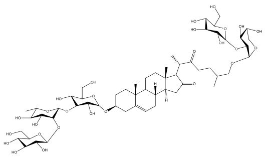	*Selaginella delicatula*	9.79±1.738 μM (IC_{50})	17.99±1.557 μM (IC_{50})	[109]
46.	Seladelicatulasine E	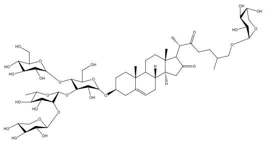	*Selaginella delicatula*	0.04±0.017 μM (IC_{50})	1.68±0.080 μM (IC_{50})	[109]
47.	Seladelicatulasine F	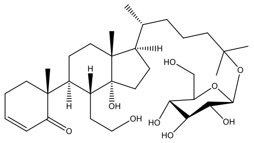	*Selaginella delicatula*	3.26±0.348 μM (IC_{50})	0.65±0.004 μM (IC_{50})	[109]
48.	Seladelicatulasine G	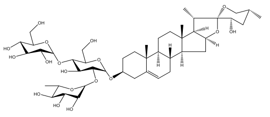	*Selaginella delicatula*	6.98±0.936 μM (IC_{50})	2.52±0.003 μM (IC_{50})	[109]
49.	Glycyrol	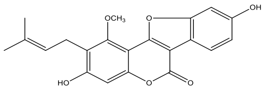	*Glycyrrhiza uralensis*	14.77±0.19 μM (IC_{50})	7.22±0.37 μM (IC_{50})	[80]
50.	Eucalyprobusal F	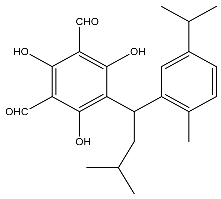	*Eucalyptus robusta*	3.22±0.36 μM (IC_{50})	-	[110]

51.	Eucalyprobusone C	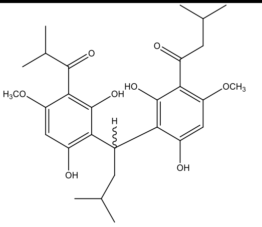	*Eucalyptus robusta*	3.82±0.22 µM (IC_{50})	-	[110]
52.	Eucalyprobusone D	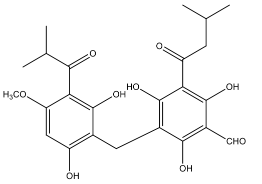	*Eucalyptus robusta*	36.22±2.29 µM (IC_{50})	-	[110]
53.	Dipolynaphthalene B	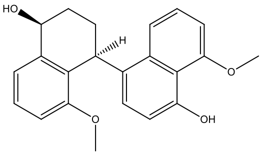	*Marasmius berteroi*	42.74±0.93% (50 µg/mL)	-	[111]
54.	Naphthone C	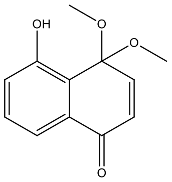	*Marasmius berteroi*	44.63±0.52% (50 µg/mL)	-	[111]
55.	Daldinone C	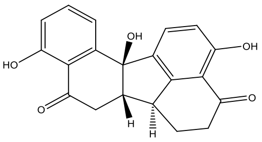	*Marasmius berteroi*	39.50±2.14% (50 µg/mL)	-	[111]
56.	8-methoxynaphthalene-1,7-diol	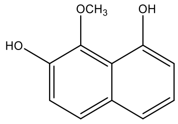	*Marasmius berteroi*	51.49±0.32% (50 µg/mL)	-	[111]
57.	Goodyschle A	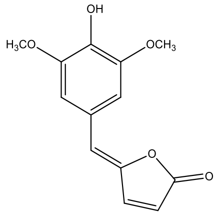	*Goodyera schlechtendaliana*	78.52±6.43 µM (IC_{50})	6.88±1.63 µM (IC_{50})	[112]
58.	(3S)-hydroxy-3′,4′-dimethoxy-L-phenylalanine	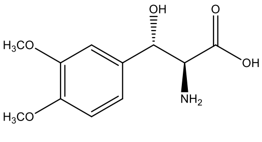	*Leucophyllum ambiguum*	1 nM (IC_{50})	-	[78]
59.	Aryl 2-benzofuran lakoochin A	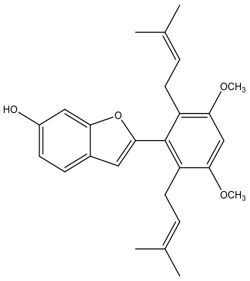	*Garcinia fusca*	27.22±0.40 µM (IC_{50})	13.65±0.05 µM (IC_{50})	[67]

60.	(6S,9R)- roseoside	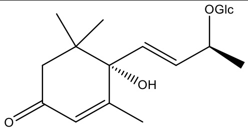	*Kadsura coccinea*	70.16±3.00 µM (IC_{50})	-	[40]
61.	sinulariapeptide A	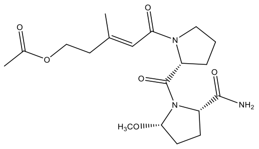	*Cochliobolus Lunatus* SCSIO41401	1.8±0.12 µM (IC_{50})	-	[113]
62.	sinulariapeptide B	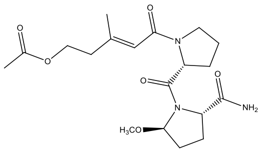	*Cochliobolus Lunatus* SCSIO41401	1.3±0.11 µM (IC_{50})	-	[113]
63.	phthalide glycerol ether	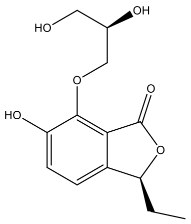	*Cochliobolus Lunatus* SCSIO41401	2.5±0.21 µM (IC_{50})	-	[113]
64.	nonacosanoic acid	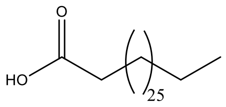	*Helichrysum plicatum*	2.58 µM (IC_{50})	3.56 µM (IC_{50})	[48]
65.	β-sitosterol-3-O-β-D-glucopyranoside	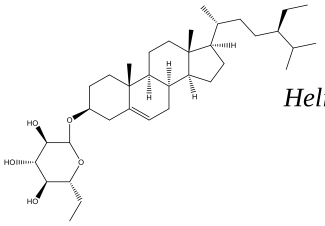	*Helichrysum plicatum*	1.72 µM (IC_{50})	1.09 µM (IC_{50})	[48]
66.	3-methylbut-3-en-1-ol-O-β-D-glucopyranoside	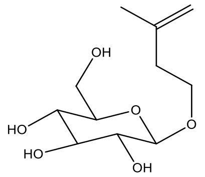	*Cupressus macrocarpa*	144.31 µg/mL (IC_{50})	-	[41]
67.	blumenol-C-glucoside	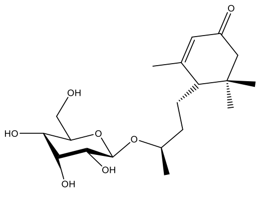	*Cupressus macrocarpa*	263.68 µg/mL (IC_{50})	-	[41]
68.	Talaromycin A	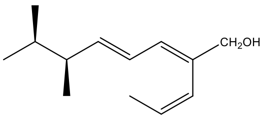	*Talaromyces aurantiacus*	12.63 µM (IC_{50})	-	[114]

69.	hyperfol C	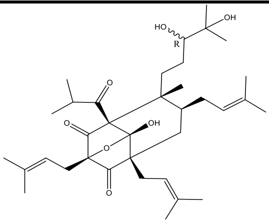	*Hypericum perforatum*	37.51±1.52 μM (IC_{50})	-	[115]
70.	hyperfol F	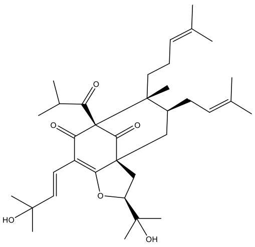	*Hypericum perforatum*	20.32±0.68 μM (IC_{50})	-	[115]
71.	hyphenrone T	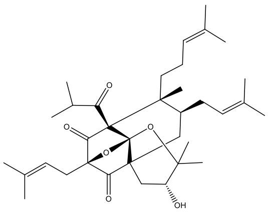	*Hypericum perforatum*	45.39±1.71 μM (IC_{50})	-	[115]
72.	Hyphenrone U	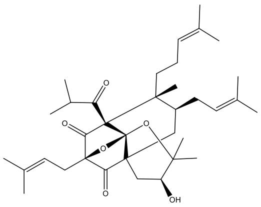	*Hypericum perforatum*	47.23±2.31 μM (IC_{50})	-	[115]
73.	Uralione K	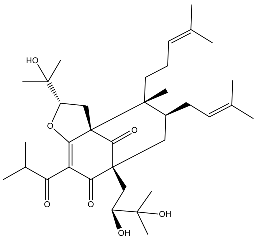	*Hypericum perforatum*	27.37±1.21 μM (IC_{50})	-	[115]
74.	cassioate D	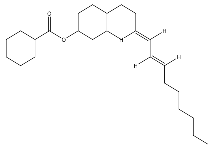	*Cassia fistula*	17.8±0.32 μM (IC_{50})	38.23±0.14 μM (IC_{50})	[116]
75.	cassioate E	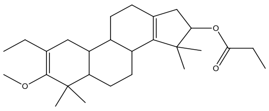	*Cassia fistula*	10.26±0.44 μM (IC_{50})	14.03±0.21 μM (IC_{50})	[116]
76.	cassioate F	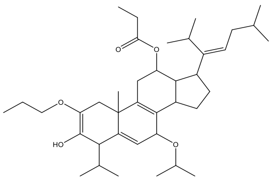	*Cassia fistula*	4.20±0.01 μM (IC_{50})	7.59±0.04 μM (IC_{50})	[116]
